# Supplementary figures and images for: International prospective observational study investigating the disease course and heterogeneity of paediatric-onset inflammatory bowel disease: the protocol of the PIBD-SETQuality inception cohort study
Source: BMJ Open. 2020 Jul 1;10(7):e035538. doi: 10.1136/bmjopen-2019-035538 (PMC7332186; doi:10.1136/bmjopen-2019-035538)

# PIBD-SETQuality Inception cohort: Flow chart biopsies

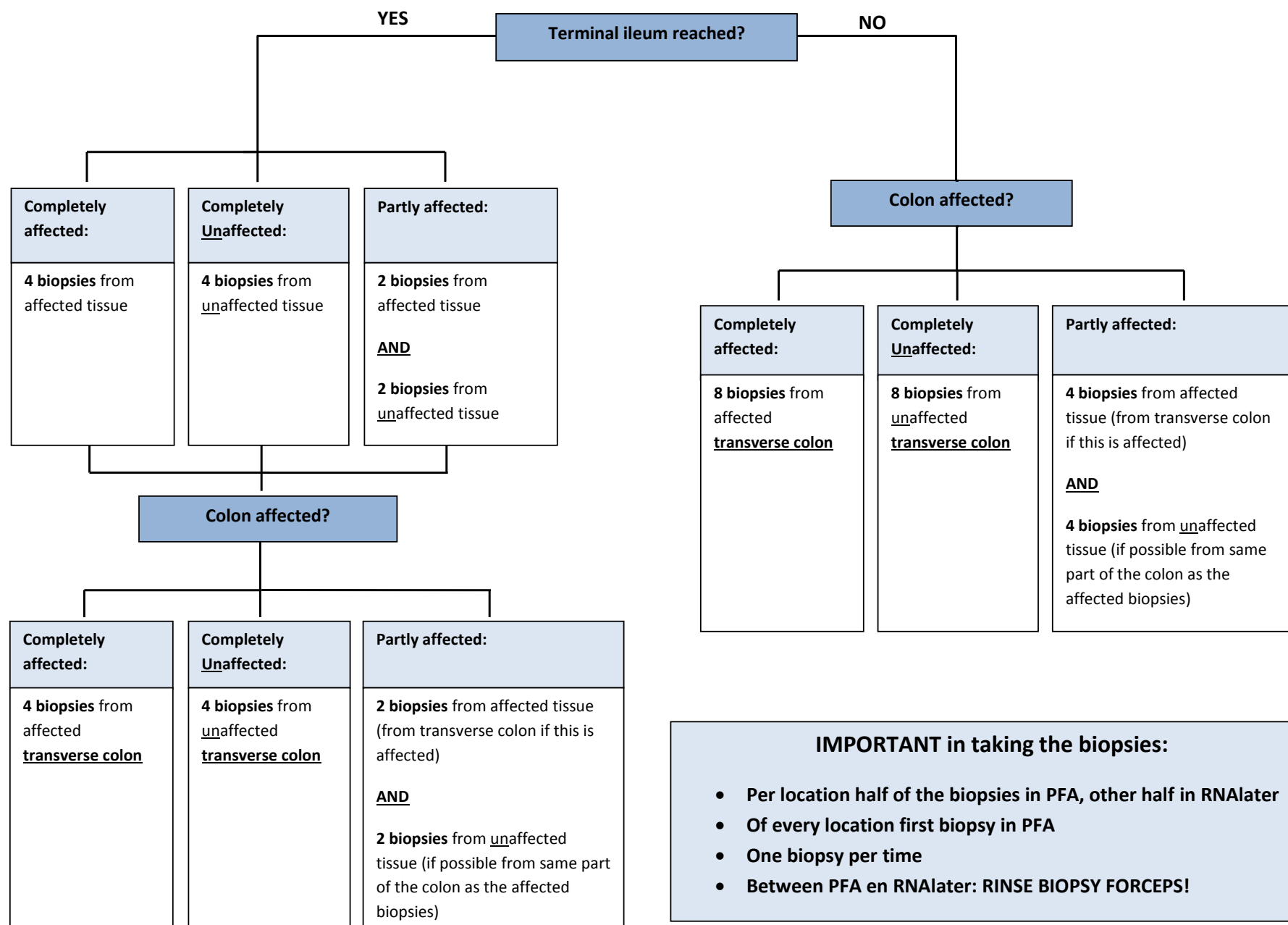

Supplement: Supplementary data [file bmjopen-2019-035538supp001.pdf]
