# Supplementary material for: International prospective observational study investigating the disease course and heterogeneity of paediatric-onset inflammatory bowel disease: the protocol of the PIBD-SETQuality inception cohort study
Source: BMJ Open. 2020 Jul 1;10(7):e035538. doi: 10.1136/bmjopen-2019-035538 (PMC7332186; doi:10.1136/bmjopen-2019-035538)
Supplement: Author's manuscript [file bmjopen-2019-035538.draft_revisions.pdf]

BMJ Open

BMJ Open is committed to open peer review. As part of this commitment we make the peer review history of every article we publish publicly available.

When an article is published we post the peer reviewers' comments and the authors' responses online. We also post the versions of the paper that were used during peer review. These are the versions that the peer review comments apply to.

The versions of the paper that follow are the versions that were submitted during the peer review process. They are not the versions of record or the final published versions. They should not be cited or distributed as the published version of this manuscript.

BMJ Open is an open access journal and the full, final, typeset and author-corrected version of record of the manuscript is available on our site with no access controls, subscription charges or pay-per-view fees (http://bmjopen.bmj.com).

If you have any questions on BMJ Open's open peer review process please email info.bmjopen@bmj.com

BMJ Open

A global prospective observational study in children and adolescents with paediatric-onset IBD: the PIBD-SETQuality inception cohort

Journal:	*BMJ Open*
Manuscript ID	bmjopen-2019-035538
Article Type:	Protocol
Date Submitted by the Author:	05-Nov-2019

Complete List of Authors:	Aardoom, Martine A; Erasmus MC Sophia Children Hospital<br>Kemos, Polychronis; Barts and The London School of Medicine and Dentistry Blizard Institute, Centre for Immunobiology<br>Tindemans, Irma; Erasmus University Medical Center-Sophia Children's Hospital, Laboratory of Pediatrics<br>Walters, Thomas D; SickKids Hospital, IBD Centre, Department of Paediatrics<br>Ruemmele, Frank; Hôpital universitaire Necker-Enfants malades, Service de Gastroentérologie pédiatrique; Université Paris Descartes<br>Samsom, Janneke N; Erasmus University Medical Center-Sophia Children's Hospital, Laboratory of Pediatrics<br>Croft, Nicholas; Barts and The London School of Medicine and Dentistry Blizard Institute, Centre for Immunobiology; Queen Mary University of London<br>de Ridder, Lissy; Erasmus MC Sophia Children Hospital, Paediatric gastroenterology

Keywords:	Inflammatory bowel disease < GASTROENTEROLOGY, Paediatric gastroenterology < GASTROENTEROLOGY, IMMUNOLOGY

SCHOLARONE™
Manuscripts

I, the Submitting Author has the right to grant and does grant on behalf of all authors of the Work (as defined in the below author licence), an exclusive licence and/or a non-exclusive licence for contributions from authors who are: i) UK Crown employees; ii) where BMJ has agreed a CC-BY licence shall apply, and/or iii) in accordance with the terms applicable for US Federal Government officers or employees acting as part of their official duties; on a worldwide, perpetual, irrevocable, royalty-free basis to BMJ Publishing Group Ltd ("BMJ") its licensees and where the relevant Journal is co-owned by BMJ to the co-owners of the Journal, to publish the Work in this journal and any other BMJ products and to exploit all rights, as set out in our licence.

The Submitting Author accepts and understands that any supply made under these terms is made by BMJ to the Submitting Author unless you are acting as an employee on behalf of your employer or a postgraduate student of an affiliated institution which is paying any applicable article publishing charge ("APC") for Open Access articles. Where the Submitting Author wishes to make the Work available on an Open Access basis (and intends to pay the relevant APC), the terms of reuse of such Open Access shall be governed by a Creative Commons licence – details of these licences and which Creative Commons licence will apply to this Work are set out in our licence referred to above.

Other than as permitted in any relevant BMJ Author's Self Archiving Policies, I confirm this Work has not been accepted for publication elsewhere, is not being considered for publication elsewhere and does not duplicate material already published. I confirm all authors consent to publication of this Work and authorise the granting of this licence.

For peer review only

Title: A global prospective observational study in children and adolescents with paediatric-onset IBD: the PIBD-SETQuality inception cohort

Martine A Aardoom[1], Polychronis Kemos[2], Irma Tindemans[3], Thomas D Walters[4], Frank M Ruemmele[5], Janneke N Samsom[3], Nicholas M Croft*[2], Lissy de Ridder*[1]; on behalf of the PIBD-SETQuality consortium and PIBD-NET

*These authors are equal senior authors.

For peer review only

COMPETING INTERESTS STATEMENT

FMR has received speaker fees from Shering-Plough, Nestlé, MeadJohnson, Ferring, MSD, Johnson & Johnson, Centocor, AbbVie; has served as a board member for SAC:DEVELOP (Johnson & Johnson), CAPE (AbbVie), LEA (AbbVie); and has been invited to MSD France, Nestlé Nutrition Institute, Nestlé Health Science, Danone, MeadJohnson, Takeda, Celgene, Biogen, Shire, Pfizer, and Therakos. LdR had collaborations (such as involved in industry sponsored studies, investigator initiated study, consultancy) with Shire, Malinckrodt, Nestlé, Celltrion, Abbvie and Pfizer; and received a grant from ZonMw, ECCO and Pfizer.

Author's affiliations

[1]Department of Paediatric Gastroenterology, The Erasmus MC Sophia Children's Hospital, Rotterdam, the Netherlands;

[2]Paediatric Gastroenterology, Centre for Immunobiology, Blizard Institute, Barts and the London School of Medicine, Queen Mary University of London, London, United Kingdom;

[3]Laboratory of Pediatrics, division Gastroenterology and Nutrition, Erasmus University Medical Center-Sophia Children's Hospital, Rotterdam, the Netherlands;

For peer review only - http://bmjopen.bmj.com/site/about/guidelines.xhtml

[4]IBD Centre, Department of Paediatrics, SickKids Hospital, University of Toronto, Toronto, Ontario, Canada;

[5]Department of Pediatric Gastroenterology, Université Paris Descartes, Sorbonne Paris Cité, APHP, Hôpital Necker Enfants Malades, Paris, France;

Corresponding author: Lissy de Ridder, MD, PhD

Address: P.O. Box 2060, 3000 CB Rotterdam, the Netherlands

E-mail: l.deridder@erasmusmc.nl, Telephone number: +31 107036049

Word count: 3192 *(The word count excludes the title page, abstract, tables, acknowledgements, contributions and references – should not exceed 4000 words)*

For peer review only - http://bmjopen.bmj.com/site/about/guidelines.xhtml

ABSTRACT

Introduction:

Paediatric Inflammatory Bowel Disease (PIBD) patients may develop a complicated disease course, including growth failure, bowel resection at young age and treatment-related adverse events all of which can have significant and lasting effects on the patient's development and quality of life. Unfortunately, we are still not able to fully explain the heterogeneity between patients and their disease course and predict which patients will respond to certain therapies or are most at risk of developing a more complicated disease course. In order to investigate this, large prospective international studies with long term follow-up are needed. Currently no such European or Asian international cohorts exist. In this first global cohort we aim to evaluate disease course and which patients are most at risk of therapy non-response or development of complicated disease based on patient and disease characteristics, immune pathology, and environmental and socio-economic factors.

Methods and analysis: In this international prospective observational study, which is part of the PIBD Network for Safety, Efficacy, Treatment and Quality improvement of care (PIBD-SETQuality), children and adolescents diagnosed with IBD <18 years are included at disease diagnosis. Follow-up is based on a visit schedule that is in line with standard PIBD care and is intended to continue for up to 20 years. Patient and disease characteristics, as well as results of investigations, are collected at baseline and during follow-up. In addition, environmental factors are being assessed. In specific centres with the ability to perform extensive immunological analyses, biomaterial is being collected and analysed in therapy naïve patients at baseline and during follow-up.

Ethics and dissemination: This study is being conducted according to the principles of the Declaration of Helsinki. Medical-ethical approval has been or will be obtained prior to patient recruitment for all sites.

Trial registration number: NCT03571373

For peer review only - http://bmjopen.bmj.com/site/about/guidelines.xhtml

Keywords: inflammatory bowel disease, Crohn's disease, ulcerative colitis, paediatrics, immunology

Word count abstract (250 – 300): 293

For peer review only

For peer review only - http://bmjopen.bmj.com/site/about/guidelines.xhtml

ARTICLE SUMMARY

Strengths and limitations of this study

- International prospective observational study with long-term follow-up examining clinical phenotype and biomarkers that may be predictive for complicated disease course, serious adverse events and response (or not) to therapy.
- Performance of extensive immunological analysis of blood and biopsies in therapy-naïve patients to evaluate heterogeneity at diagnosis and during follow-up based on underlying immune dysfunction.
- The first prospective PIBD cohort that includes patients from several European and some Asian countries, which enables comparison of therapeutic strategies between countries and continents, and outcomes of these strategies.
- Large scale-collection of quality of life measures to correlate to clinical findings and compare this across countries and ethnicities.
- Different methods between centres for measurement of laboratory parameters that are part of standard clinical care serve as a limitation to this study.

For peer review only

For peer review only - http://bmjopen.bmj.com/site/about/guidelines.xhtml

INTRODUCTION

Background

Paediatric inflammatory bowel disease (PIBD) is a chronic disease that often leads to disabling symptoms such as abdominal pain, diarrhoea and rectal bleeding. Inflammatory bowel disease (IBD) comprises Crohn's disease (CD), ulcerative colitis (UC) and IBD-unclassified (IBD-U). Although IBD is most frequently known as an adult disease, in 5-25% of cases it is diagnosed during childhood or adolescence.[1-3] The incidence of IBD in Western Europe and North-America ranges from 1.85 to 23.82 per 100,000 for CD and 1.9 to 23.14 per 100,000 for UC.[4] Despite the fact that the incidence of IBD varies among different countries, a current concern is that the general trend shows increasing incidence rates during the last decades, especially among the young and very young patients <10 years of age.[1 3 5-7] Compared to IBD with adult-onset, PIBD reflects a more severe disease.[8-10] Consequences of the disease such as growth failure and bowel resection at a young age may have a large impact on the patient's further development and quality of life. In addition, the early onset of this disease regularly leads to an early use of intensive therapies with a life-long risk of treatment-related adverse events.[8 11]

The pathogenesis of PIBD is currently partly explained by a combination of a genetic predisposition, microbial factors and a susceptibility of the immune system leading to an aberrant inflammatory immune response.[12-14] Treatment strategies therefore focus on modulating or suppressing the immune response by immunosuppressive drugs or biologicals. Despite the known heterogeneity within both paediatric CD and UC patients at disease diagnosis and during follow-up, we are still not able to predict which patients are at risk of developing a complicated disease course and which patients will respond to therapy.[15 16] Therefore, the majority of PIBD patients have been treated following the same step-up approach, a strategy in which patients start with a therapy at the bottom of the therapeutic pyramid and medications at the top are considered more potent but may present greater risk to the patient. This may lead to a delay in treatment response and increases the risk of ongoing inflammation risking penetrating and stricturing complications. The ability to predict complicated

For peer review only - http://bmjopen.bmj.com/site/about/guidelines.xhtml

disease course and response or non-response to therapy would be of immense value. This is essential to develop strategies that balance, on an individual basis, therapeutic effectiveness with risks of treatment.

In studies including adult IBD patients, several clinical risk factors have been identified for the development of a complicated disease course in Crohn's disease and the need for colectomy in UC patients.[17 18] Given the differences in disease phenotype, course of disease and benefits and risks of treatment between children and adults, findings from adult studies do not directly apply to PIBD. Few studies have assessed risk factors in paediatric-onset CD patients and found that both stricturing disease behaviour[15 19 20] and older age at diagnosis [21-23] are associated with an increased risk for the need for surgery. In paediatric-onset UC patients the PUCAI at diagnosis and 3 months after diagnosis is found to be an essential predictor of colectomy.[24 25] Kugathasan et al. demonstrated that early anti-TNF therapy was associated with a decreased risk of penetrating but not stricturing complications.[26]

Due to the small number of available studies comprising a large variety of data on several different outcomes and predictors combined with a mainly retrospective set-up, to date most findings regarding predictors of disease course in PIBD are inconclusive. The majority of the identified predictors are demographic or associated with the disease phenotype and studies lack findings on the predictive value of biomarkers. Despite the known role of the immune system in PIBD, no immunological biomarkers have been identified to correlate with the disease phenotype or disease course. Therefore, there is an urgent need to generate a prospective long-term real-world cohort designed to analyse effectiveness and safety signals with the ability to correlate them to individual risk factors in well-phenotyped patients. To address this issue, the Paediatric Inflammatory Bowel Diseases Network for Safety, Efficacy, Treatment and Quality (PIBD-SETQuality) inception cohort was designed. Besides a few prospective PIBD cohorts, established in the United States and Canada, no international European cohorts currently exist to assess this.[26] Due to possible differences in genotype, environmental influences and treatment algorithms, European data are required. With this paper, we aim to inform the IBD

For peer review only - http://bmjopen.bmj.com/site/about/guidelines.xhtml

research community about the existence of the PIBD-SETQuality inception cohort and provide insight into the establishment of this cohort.

Objectives

PIBD-SETQuality is an international project with the overall goal to develop and validate a treatment algorithm for PIBD based on high or low-risk predictors for early complicated or relapsing disease. In this inception cohort, predictors are identified through prospective collection of longitudinal PIBD data in the first European cohort. Real-world longitudinal data with preferably a 20-year follow-up period is collected to examine effectiveness of existing treatment strategies and identify treatment or disease-related risks. The objective is to search for factors predictive for a complicated disease course, specific serious adverse events (SAEs) and predictors of response or non-response to therapy (Table 1).

For peer review only - http://bmjopen.bmj.com/site/about/guidelines.xhtml

Potentially relevant prognostic factors	
**1**	Family history
**2**	Medical history
**3**	Ethnicity
**4**	Severity of disease at diagnosis
**5**	Disease localization
**6**	Course of disease
**7**	Level of inflammatory markers
**8**	Faecal calprotectin level
**9**	Endoscopic findings
**10**	Immunological biomarkers
**11**	Genetic status
**12**	Environmental factors
**13**	Diet
**14**	Health economic status
**15**	Psychosocial status

Table 1. Potentially relevant prognostic factors in prediction of disease course and therapy responsiveness in PIBD

For peer review only - http://bmjopen.bmj.com/site/about/guidelines.xhtml

In addition, this cohort enables investigation of PIBD heterogeneity based on immunological biomarkers and racial or environmental factors and evaluation of international differences in treatment strategies.

METHODS AND ANALYSIS

Study design

The PIBD-SETQuality inception cohort is a multicentre prospective observational study in patients with PIBD. Participants are followed from the moment of diagnosis up to 20 years thereafter. During the first year after diagnosis data collection is performed more frequently and reduced to annual visits after the second year of follow-up (Table 2).

For peer review only - http://bmjopen.bmj.com/site/about/guidelines.xhtml

Visit number	Visit 0	Visit 1	Visit 2	Visit 3	Visit 4	Visit 5	Annual visits	Unscheduled visit
**Time after start of therapy**	**Prior to start of therapy**	**4 weeks after start of therapy**	**3 months**	**6 months**	**12 months**	**18 months**	**24, 36 months, etc.**	
**Activity**								
Study explained informed consent	X							
Collection of routine clinical and laboratory data	X	X	X	X	X	X	X	X
Extra blood sample taken at time of routine blood draw (maximum of 20 ml)*	O	O	O	O	O	O	O	O
Extra biopsies taken at time of clinically required colonoscopy (maximum of 8)*	O							O
Tissue sample in case of indication for surgical resection*		O	O	O	O	O	O	O
Environmental questionnaire	X							
IMPACT III & EQ-5D questionnaires	X		X (EQ-5D)	X (EQ-5D)	X (both)	X (EQ-5D)	X (both)	
School attendance and WPAI questionnaires	X (both)		X (both)		X (both)		X (both)	

Table 2. Visit schedule and included activities for PIBD-SETQ inception cohort.
*These activities will only be performed in patients included in the subcohort. ''X'' is performed in all patients, ''O'' is only performed in subcohort patient

For peer review only - http://bmjopen.bmj.com/site/about/guidelines.xhtml

At least 500 PIBD patients need to be included in the inception cohort. However, since this is an observational study, depending on the feasibility, the study will aim to recruit 900 patients. In specific centres with the capacity to perform immunological analyses participants are included in a subcohort for additional collection of biological specimens. This allows in depth characterization of immunological pathways in 100 CD patients and 50 UC patients, and provides the opportunity to relate predictive factors to underlying immune dysfunction during follow-up. The inception cohort is part of the PIBD-SETQuality project, which is funded by Horizon 2020.

Eligibility criteria

Children and adolescents <18 years with a likely or confirmed diagnosis of IBD are eligible. Diagnosis has to be made or confirmed within the first 2 months after inclusion. Diagnosis must be based on history, physical examination, laboratory, endoscopic, radiological and histological features according to the revised Porto criteria.[27] If the diagnosis IBD is not confirmed after the investigations are complete the patient will be excluded from follow-up. Other inclusion criteria comprise available data on all diagnostic procedures for inclusion in the database, informed consent of patient and parents according to the national guidelines, and, in case of inclusion in the subcohort for collection of biomaterial, the patient has not started therapy yet at the moment of diagnostic endoscopy. Patients are excluded from this study 1) when they are on similar treatments as for IBD but for other conditions 2) when they are known to have conditions directly affecting IBD (e.g. immunodeficiency or major gastrointestinal resections) and 3) in case of inability to read or understand the patient and family information sheets.

Recruitment and data collection

All eligible patients are asked for informed consent to participate in the inception cohort. Within this study clinical data and results of questionnaires will be collected in all patients. Biomaterial is

For peer review only - http://bmjopen.bmj.com/site/about/guidelines.xhtml

collected as part of the subcohort in specific centres with the ability to perform immunological analyses.

Clinical data

At baseline, data on demographics, family history, diagnosis, disease activity, disease location and results of physical examination, endoscopy, radiographic imaging and laboratory results are collected. Validated scores and classifications such as the weighted Paediatric Crohn's Disease Activity Index (wPCDAI), Paediatric Ulcerative Colitis Activity Index (PUCAI), Simple Endoscopic Score for Crohn's Disease (SES-CD) and Ulcerative Colitis Endoscopic Index of Severity (UCEIS) are being used.[28-31] During follow-up the clinical disease activity scores, results of additional investigations and detailed treatment information are collected at fixed time points and hospitalisations.

Questionnaires

To assess the quality of life (QoL) the validated IMPACT III questionnaire is being used. This questionnaire is a disease specific health-related assessment of QoL, divided in the domains emotional functioning, social functioning, body image, and well-being.[32 33] Validated EQ-5D questionnaires are being used to assess the health status of the patient. The questionnaire consists of five dimensions; mobility, self-care, usual activities, pain/discomfort and anxiety/depression.[34] Parent's work productivity is assessed by the Work Productivity and Activity Impairment questionnaire (WPAI). Due to the lack of validated questionnaires assessing the child's school attendance, a non-validated questionnaire is being used to address this subject. The frequency of the assessment of these questionnaires is shown in Table 2. At baseline, many environmental factors are being assessed comprising e.g. parent's smoking behaviour, dietary factors, antibiotic use, sun exposure, previous enteritis and appendectomy.

Biomaterial

For peer review only - http://bmjopen.bmj.com/site/about/guidelines.xhtml

In several centres biological specimens is collected in addition to the clinical data. When informed consent for the collection of biomaterial is obtained, blood samples and intestinal biopsies from affected and non-affected tissue are collected prior to treatment initiation and during follow-up visits in concurrence with routine clinical diagnostics. Analyses of biopsies include immunohistochemistry, targeted quantitative mRNA analyses and unbiased mRNA sequencing analyses. Peripheral blood samples are collected for plasma proteome analyses, plasma antimicrobial reactivity, in depth phenotypic and functional leukocyte analyses and screening for genetic polymorphisms (Figure 1). Immunological analyses will be related to the previously described clinical data that is being collected. Patients enrolled in the subcohort in whom the diagnosis of IBD is not confirmed after additional investigations will be analysed as non-IBD controls.

Outcome measures

This study design and statistical analysis plan are based on one primary and several secondary and exploratory outcomes. At baseline, prior to the start of therapy, the clinical characteristics of PIBD patients will be reviewed and compared by country, as well as racial background, in children with active disease. Immunological biomarkers in therapy naïve patients are reviewed in order to classify disease heterogeneity based on underlying immune pathology. Thereto, immunological parameters will be related to clinical and morphological disease features at this time point.

The primary outcome is clinical remission at one year, which reflects our aim to study therapy response and non-response. Clinical remission is defined as a wPCDAI <12.5 in CD patients and a PUCAI <10 in UC or IBDU patients. According to the set hypothesis, the disease location, extension, behaviour and type of induction therapy are important factors that influence the primary outcome while age at diagnosis and the initial disease activity score are expected to be important covariates. In addition, biochemical and immunological parameters at baseline will be evaluated to predict clinical remission. In a sub-analysis the effect of earlier use of

For peer review only - http://bmjopen.bmj.com/site/about/guidelines.xhtml

immunomodulators and biologics (less than 3 months from diagnosis) on remission rates will be investigated by comparing groups with early and later use.

One of the secondary outcomes of this study is to assess clinical remission over a period of 3 and 6 months since start of treatment to evaluate short-term response. In addition, the need for treatment intensification within one year will be assessed. The longitudinal follow-up analyses of individual immunological biomarkers will reveal which immune parameters change when patients are in clinical remission. Clinical, biochemical and immunological findings at baseline and 12 weeks after start of therapy will be assessed for their predictive value on the disease course after one, three, five, 10, 15 and 20 years of follow-up. Lastly, as a longer-term secondary outcome we will assess 1) moderate or severe disease over the last 6-12 months, 2) the development of complications such as fibrostricturing disease, penetrating disease, active perianal fistula or an abscess, 3) the need for surgery, and 4) the need for biologic use or need for treatment intensification, after one, three, five, 10, 15 and 20 years.

As an explanatory outcome, the initial management of newly diagnosed PIBD patients will be evaluated. The type of induction therapy and proportion of patients started on early immunomodulators or anti-TNF agents will be assessed, and compared by country or region. Several environmental factors will be evaluated to assess the possible role in the heterogeneity within presenting phenotype as well as disease course. Lastly, longitudinal findings on QoL, health status, parents' work productivity and child's school attendance will be evaluated, compared by country or region and correlated to disease activity and disease course.

For peer review only

Sample size calculation

To ensure that the study is adequately powered for the primary outcome, we have calculated the minimum required sample size, which we have also adjusted for a 10% expected information loss and multiple comparisons. To maintain the global significance level alpha at 0.05, for the sample size calculations, we decreased the significance level for the univariate analyses to 0.01 per

For peer review only - http://bmjopen.bmj.com/site/about/guidelines.xhtml

Bonferroni correction. As we aim to capture predictors with significant influence on the remission, we set the effect size of the primary outcome at 25%. In order to detect a 25% or higher difference between the compared groups, as defined by disease characteristics and therapy induction types and considering the variable sampling ratio (2:1 to 1:2 depending on the factor), we will need 203 CD patients and 214 UC/IBDU patients. Considering approximately 40% of PIBD diagnoses comprises UC/IBDU, we will need 535 PIBD patients. Subsequently, the CD group will have slightly increased power than the originally planned 80%.

Data collection and management

Data are collected in REDCap, a secured database, by using online case report forms (eCRF). CRFs are based on the dataset of the Canadian inception cohort by the Canadian Children Inflammatory Bowel Disease Network (CIDsCaNN) and adjusted to the needs of this study. A monitor performs remote (digital) monitoring for each participating centre yearly after inclusion. The coordinating investigator runs consistency checks on a monthly basis and produces queries to be resolved by the local investigators. To secure accurate comparison of biomarkers in tissue specimens obtained from different centres, highly reproducible sample preparation is required across all centres participating in this study. Therefore, all aspects of sample acquisition and all reagents will be strictly regulated and sample quality will be tightly monitored.

Analysis and statistical methods

Data analysis will be performed with SPSS version 26 or higher for Windows (IBB SPSS, Chicago, Illinois, USA) and R version 3.6 or higher (R Foundation for Statistical Computing, Vienna, Austria). Descriptive statistics will be computed overall and per disease (CD and UC). Phenotypic grouping will be performed according to specific disease characteristics (e.g. age of onset, disease localization, disease behaviour). Next to this, patients will be categorised according to treatment categories. The appropriate descriptive statistics will be used to summarize the demographic characteristics. The primary outcome will be analysed using a 2-

For peer review only - http://bmjopen.bmj.com/site/about/guidelines.xhtml

proportion Z test for the comparison of the different groups in our sample, using a stricter confidence level of 0.01 per Bonferroni correction. All proportions will be summarised per group and 95% confidence intervals will be provided. The important factors from the univariate analysis will be used to build a generalised linear model with a logit link function. This will be a multiple analysis of the effects that the selected factors and covariates have on the outcome of clinical remission. The final model will also include interaction terms, if necessary, and will be optimised based on fit diagnostics and residual analysis. The same approach will be used for the secondary outcomes of clinical remission. We will use a mixed-effects linear model to study the effects of the predictors on the disease activity index over time, taking into account the non-independence between the observations due to the repeated measurements from each patient. For the additional long-term secondary outcomes that have a time-to-event nature, we will use Kaplan-Meier curves to summarise the effects of the categorical factors on the outcome. This will be used to build a Cox-regression model with the important factors and covariates that have a significant effect on the time and frequency of the events as defined in the outcomes section. Similar methods will be used for the analysis of the exploratory outcomes. For these outcomes, propensity scores and multivariate methods are required, in particular for the immunological data, including principal component, factor and cluster analysis. Incidences of SAEs and the SAE rate per 100 patient years will be calculated.

For peer review only

Study status

The first study participant was recruited in 2017. Enrolment is expected to be completed by the end of 2021.

Patient and Public Involvement

Patients were not involved in the development of this study; however, the French patient charity AFA Crohn, RCH, France, was involved in the study design and critically reviewed and commented upon main aspects of the trial.

For peer review only - http://bmjopen.bmj.com/site/about/guidelines.xhtml

Discussion

The PIBD-SETQuality inception cohort is a unique study, being the first cohort in its size including PIBD patients from European and Asian countries. The homogeneous collection of data from several different countries in one cohort enables comparison of disease phenotype and treatment paradigms between countries and continents. The hypothesised role of environmental factors in the pathogenesis of PIBD, might thus be assessed within this cohort. In addition, being a cohort with real-world data, this study will complement data derived from clinical trials and provide insight in drug use in everyday practice and the related international differences.

The main outcome of this study will be the prediction of a complicated disease course and response or non-response to therapy. One of the important strengths of this study is the collection of biomaterial in therapy naïve patients. Studies assessing immunological biomarkers in PIBD patients are scarce, and even lacking in therapy naïve patients. The prospective set-up of this study will reveal whether immunological biomarkers in PIBD change overtime and in response to therapy. In addition, these biomarkers can be correlated to the clinical data. Besides finding predictive factors of a complicated disease course and response to therapy within this real-world cohort, we will also use this cohort to examine and possibly validate previously reported findings from other studies such as the GROWTH CD study (NCT00711945).

ETHICS AND DISSEMINATION

The study is being conducted according to the principles of the Declaration of Helsinki and so far has been approved by the ethical committees of Erasmus Medical Centre Rotterdam in the Netherlands, the National Health Service (NHS) authorities in England and ethical committees of participating centres in Italy, Israel, France, United Arabic Emirates, Malaysia and Japan. Handling of patient material complies with the legislation of the relevant country and the

For peer review only

For peer review only - http://bmjopen.bmj.com/site/about/guidelines.xhtml

European General Data Protection Regulation (GDPR). In accordance with the H2020 general grant agreement, the dissemination process will ensure open access to the peer-reviewed scientific publications resulting from this project and the related bibliographic meta-data.

ACKNOWLEDGEMENTS

We would like to thank all patients who participate in this study; all nurses who are participating in data entry and all the gastroenterologists who are involved in patient enrolment. Collaborating sites and principal investigators ordered by country:

England: N. Croft, Royal London Hospital, London; F. Cameron, Alder Hey Children's NHS Foundation, Liverpool; P. Henderson, Royal Hospital for Sick Children, Edinburgh; J. Ashton, University Hospital Southampton NHS Foundation Trust, Southampton; R. Russell, The Royal Hospital for Children Glasgow, Glasgow; M. Rafeeq, Birmingham children's hospital, Birmingham; N. Nedelkopoulou and P. Rao, Sheffield Children's Hospital, Sheffield; A. Rodrigues, Oxford Children's Hospital, Oxford; J. Fell, Chelsea and Westminster Hospital NHS Foundation Trust, London; D. Devadason, Nottingham Children's Hospital, Nottingham; J. Hart, Royal Devon & Exeter NHS Foundation Trust, Exeter; J. Cohen, University College London Hospitals NHS Foundation Trust, London;

The Netherlands: L. de Ridder, Erasmus Medical Center, Rotterdam; T. Hummel, Medisch Spectrum Twente, Enschede; M. Wessels, Rijnstate, Arnhem; C. van der Feen, Jeroen Bosch hospital; 's Hertogenbosch;

Israel: A. Levine, Wolfson Medical Center, Holon; D. Turner, Shaare Zedek Medical Center, Jerusalem; A. Assa, Schneider Children's Medical Center of Israel, Petach-Tikva

Italy: M. Aloi, Sapienza University, Rome;

France: F. Ruemmele, Hôpital Necker Enfants Malades, Paris;

Japan: K. Arai, National Center for Child Health and Development, Tokyo;

For peer review only - http://bmjopen.bmj.com/site/about/guidelines.xhtml

Malaysia: WS Lee, University Malay Medical Center, Kuala Lumpur;

United Arabic Emirates: C. Tzivinikos, Al Jalila Children's Specialty Hospital, Dubai

AUTHOR'S CONTRIBUTIONS

MAA, PK, TW, FMR, JNS, NMC and LdR were involved in the protocol design. All authors are involved in the implementation of this study. All authors contributed to drafting the manuscript and provided important intellectual content.

FUNDING STATEMENT

This project is a Pediatric Inflammatory Bowel Disease Network (PIBD-NET) initiative. It was supported as part of the PIBD-SETQuality project funded by the European Commission Horizon 2020, funding source number 668023. The study sponsor is PIBD-NET.

For peer review only

For peer review only - http://bmjopen.bmj.com/site/about/guidelines.xhtml

FIGURE LEGEND

Figure 1. Different immunological analyses that will be performed. Data will be correlated to the clinical data. Abbreviations: IHC, immunohistochemistry; ISH, in situ hybridization.

For peer review only

For peer review only - http://bmjopen.bmj.com/site/about/guidelines.xhtml

REFERENCES

1. Benchimol EI, Fortinsky KJ, Gozdyra P, et al. Epidemiology of pediatric inflammatory bowel disease: a systematic review of international trends. *Inflamm Bowel Dis* 2011;**17**(1):423-39.
2. Gower-Rousseau C, Vasseur F, Fumery M, et al. Epidemiology of inflammatory bowel diseases: new insights from a French population-based registry (EPIMAD). *Dig Liver Dis* 2013;**45**(2):89-94.
3. Ghione S, Sarter H, Fumery M, et al. Dramatic Increase in Incidence of Ulcerative Colitis and Crohn's Disease (1988-2011): A Population-Based Study of French Adolescents. *Am J Gastroenterol* 2018;**113**(2):265-72.
4. Ng SC, Shi HY, Hamidi N, et al. Worldwide incidence and prevalence of inflammatory bowel disease in the 21st century: a systematic review of population-based studies. *Lancet* 2018;**390**(10114):2769-78.
5. Coughlan A, Wylde R, Lafferty L, et al. A rising incidence and poorer male outcomes characterise early onset paediatric inflammatory bowel disease. *Aliment Pharmacol Ther* 2017;**45**(12):1534-41.
6. Bequet E, Sarter H, Fumery M, et al. Incidence and Phenotype at Diagnosis of Very-early-onset Compared with Later-onset Paediatric Inflammatory Bowel Disease: A Population-based Study [1988-2011]. *J Crohns Colitis* 2017;**11**(5):519-26.
7. Sykora J, Pomahacova R, Kreslova M, et al. Current global trends in the incidence of pediatric-onset inflammatory bowel disease. *World J Gastroenterol* 2018;**24**(25):2741-63.
8. Duricova D, Burisch J, Jess T, et al. Age-related differences in presentation and course of inflammatory bowel disease: an update on the population-based literature. *J Crohns Colitis* 2014;**8**(11):1351-61.
9. Duricova D, Fumery M, Annese V, et al. The natural history of Crohn's disease in children: a review of population-based studies. *Eur J Gastroenterol Hepatol* 2017;**29**(2):125-34.
10. Van Limbergen J, Russell RK, Drummond HE, et al. Definition of phenotypic characteristics of childhood-onset inflammatory bowel disease. *Gastroenterology* 2008;**135**(4):1114-22.
11. Jess T, Rungoe C, Peyrin-Biroulet L. Risk of colorectal cancer in patients with ulcerative colitis: a meta-analysis of population-based cohort studies. *Clin Gastroenterol Hepatol* 2012;**10**(6):639-45.
12. Kaser A, Zeissig S, Blumberg RS. Inflammatory bowel disease. *Annu Rev Immunol* 2010;**28**:573-621.
13. Jostins L, Ripke S, Weersma RK, et al. Host-microbe interactions have shaped the genetic architecture of inflammatory bowel disease. *Nature* 2012;**491**(7422):119-24.
14. Henderson P, van Limbergen JE, Wilson DC, et al. Genetics of childhood-onset inflammatory bowel disease. *Inflamm Bowel Dis* 2011;**17**(1):346-61.
15. Vernier-Massouille G, Balde M, Salleron J, et al. Natural history of pediatric Crohn's disease: a population-based cohort study. *Gastroenterology* 2008;**135**(4):1106-13.
16. Gower-Rousseau C, Dauchet L, Vernier-Massouille G, et al. The natural history of pediatric ulcerative colitis: a population-based cohort study. *Am J Gastroenterol* 2009;**104**(8):2080-8.
17. Torres J, Caprioli F, Katsanos KH, et al. Predicting Outcomes to Optimize Disease Management in Inflammatory Bowel Diseases. *J Crohns Colitis* 2016;**10**(12):1385-94.
18. Lakatos PL. Prediction of disease course in inflammatory bowel diseases. *World J Gastroenterol* 2010;**16**(21):2589-90.
19. De Greef E, Mahachie John JM, Hoffman I, et al. Profile of pediatric Crohn's disease in Belgium. *J Crohns Colitis* 2013;**7**(11):e588-98.
20. Rinawi F, Assa A, Hartman C, et al. Incidence of Bowel Surgery and Associated Risk Factors in Pediatric-Onset Crohn's Disease. *Inflamm Bowel Dis* 2016;**22**(12):2917-23.
21. Gupta N, Cohen SA, Bostrom AG, et al. Risk factors for initial surgery in pediatric patients with Crohn's disease. *Gastroenterology* 2006;**130**(4):1069-77.

For peer review only - http://bmjopen.bmj.com/site/about/guidelines.xhtml

22. Henderson P, Kennedy NA, Van Limbergen JE, et al. Serum C-reactive protein and CRP genotype in pediatric inflammatory bowel disease: influence on phenotype, natural history, and response to therapy. *Inflamm Bowel Dis* 2015;**21**(3):596-605.
23. Schaefer ME, Machan JT, Kawatu D, et al. Factors that determine risk for surgery in pediatric patients with Crohn's disease. *Clin Gastroenterol Hepatol* 2010;**8**(9):789-94.
24. Rinawi F, Assa A, Eliakim R, et al. Risk of Colectomy in Patients With Pediatric-onset Ulcerative Colitis. *J Pediatr Gastroenterol Nutr* 2017;**65**(4):410-15.
25. Schechter A, Griffiths C, Gana JC, et al. Early endoscopic, laboratory and clinical predictors of poor disease course in paediatric ulcerative colitis. *Gut* 2015;**64**(4):580-8.
26. Kugathasan S, Denson LA, Walters TD, et al. Prediction of complicated disease course for children newly diagnosed with Crohn's disease: a multicentre inception cohort study. *Lancet* 2017;**389**(10080):1710-18.
27. Levine A, Koletzko S, Turner D, et al. ESPGHAN revised porto criteria for the diagnosis of inflammatory bowel disease in children and adolescents. *J Pediatr Gastroenterol Nutr* 2014;**58**(6):795-806.
28. Turner D, Otley AR, Mack D, et al. Development, validation, and evaluation of a pediatric ulcerative colitis activity index: a prospective multicenter study. *Gastroenterology* 2007;**133**(2):423-32.
29. Hyams J, Markowitz J, Otley A, et al. Evaluation of the pediatric crohn disease activity index: a prospective multicenter experience. *J Pediatr Gastroenterol Nutr* 2005;**41**(4):416-21.
30. Daperno M, D'Haens G, Van Assche G, et al. Development and validation of a new, simplified endoscopic activity score for Crohn's disease: the SES-CD. *Gastrointest Endosc* 2004;**60**(4):505-12.
31. Travis SP, Schnell D, Krzeski P, et al. Developing an instrument to assess the endoscopic severity of ulcerative colitis: the Ulcerative Colitis Endoscopic Index of Severity (UCEIS). *Gut* 2012;**61**(4):535-42.
32. Werner H, Landolt MA, Buehr P, et al. Validation of the IMPACT-III quality of life questionnaire in Swiss children with inflammatory bowel disease. *J Crohns Colitis* 2014;**8**(7):641-8.
33. Abdovic S, Mocic Pavic A, Milosevic M, et al. The IMPACT-III (HR) questionnaire: a valid measure of health-related quality of life in Croatian children with inflammatory bowel disease. *J Crohns Colitis* 2013;**7**(11):908-15.
34. Herdman M, Gudex C, Lloyd A, et al. Development and preliminary testing of the new five-level version of EQ-5D (EQ-5D-5L). *Qual Life Res* 2011;**20**(10):1727-36.

For peer review only

For peer review only - http://bmjopen.bmj.com/site/about/guidelines.xhtml

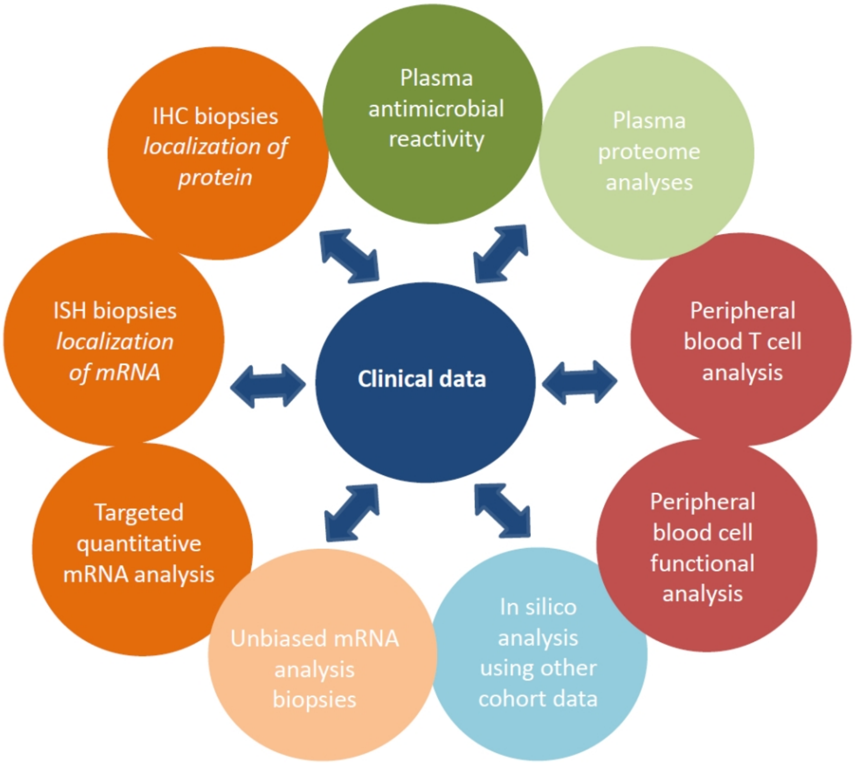

Different immunological analyses that will be performed. Data will be correlated to the clinical data. Abbreviations: IHC, immunohistochemistry; ISH, in situ hybridization.

STROBE 2007 (v4) Statement—Checklist of items that should be included in reports of *cohort studies*

Section/Topic	Item #	Recommendation	Reported on page #
**Title and abstract**	1	(*a*) Indicate the study's design with a commonly used term in the title or the abstract	
		(*b*) Provide in the abstract an informative and balanced summary of what was done and what was found	
**Introduction**			
Background/rationale	2	Explain the scientific background and rationale for the investigation being reported	
Objectives	3	State specific objectives, including any prespecified hypotheses	
**Methods**			
Study design	4	Present key elements of study design early in the paper	
Setting	5	Describe the setting, locations, and relevant dates, including periods of recruitment, exposure, follow-up, and data collection	
Participants	6	(*a*) Give the eligibility criteria, and the sources and methods of selection of participants. Describe methods of follow-up	
		(*b*) For matched studies, give matching criteria and number of exposed and unexposed	
Variables	7	Clearly define all outcomes, exposures, predictors, potential confounders, and effect modifiers. Give diagnostic criteria, if applicable	
Data sources/ measurement	8*	For each variable of interest, give sources of data and details of methods of assessment (measurement). Describe comparability of assessment methods if there is more than one group	
Bias	9	Describe any efforts to address potential sources of bias	
Study size	10	Explain how the study size was arrived at	
Quantitative variables	11	Explain how quantitative variables were handled in the analyses. If applicable, describe which groupings were chosen and why	
Statistical methods	12	(*a*) Describe all statistical methods, including those used to control for confounding	
		(*b*) Describe any methods used to examine subgroups and interactions	
		(*c*) Explain how missing data were addressed	
		(*d*) If applicable, explain how loss to follow-up was addressed	
		(*e*) Describe any sensitivity analyses	
**Results**			

For peer review only

Participants	13*	(a) Report numbers of individuals at each stage of study—eg numbers potentially eligible, examined for eligibility, confirmed eligible, included in the study, completing follow-up, and analysed	
		(b) Give reasons for non-participation at each stage	
		(c) Consider use of a flow diagram	
Descriptive data	14*	(a) Give characteristics of study participants (eg demographic, clinical, social) and information on exposures and potential confounders	
		(b) Indicate number of participants with missing data for each variable of interest	
		(c) Summarise follow-up time (eg, average and total amount)	
Outcome data	15*	Report numbers of outcome events or summary measures over time	
Main results	16	(*a*) Give unadjusted estimates and, if applicable, confounder-adjusted estimates and their precision (eg, 95% confidence interval). Make clear which confounders were adjusted for and why they were included	
		(*b*) Report category boundaries when continuous variables were categorized	
		(*c*) If relevant, consider translating estimates of relative risk into absolute risk for a meaningful time period	
Other analyses	17	Report other analyses done—eg analyses of subgroups and interactions, and sensitivity analyses	
**Discussion**			
Key results	18	Summarise key results with reference to study objectives	
**Limitations**			
Interpretation	20	Give a cautious overall interpretation of results considering objectives, limitations, multiplicity of analyses, results from similar studies, and other relevant evidence	
Generalisability	21	Discuss the generalisability (external validity) of the study results	
**Other information**			
Funding	22	Give the source of funding and the role of the funders for the present study and, if applicable, for the original study on which the present article is based	

*Give information separately for cases and controls in case-control studies and, if applicable, for exposed and unexposed groups in cohort and cross-sectional studies.

Note: An Explanation and Elaboration article discusses each checklist item and gives methodological background and published examples of transparent reporting. The STROBE checklist is best used in conjunction with this article (freely available on the Web sites of PLoS Medicine at http://www.plosmedicine.org/, Annals of Internal Medicine at http://www.annals.org/, and Epidemiology at http://www.epidem.com/). Information on the STROBE Initiative is available at www.strobe-statement.org.

For peer review only

BMJ Open

An international prospective observational study investigating the disease course and heterogeneity of paediatric-onset inflammatory bowel disease: the protocol of the PIBD-SETQuality inception cohort study

Journal:	*BMJ Open*
Manuscript ID	bmjopen-2019-035538.R1
Article Type:	Protocol
Date Submitted by the Author:	19-Mar-2020
Complete List of Authors:	Aardoom, Martine A; Erasmus MC Sophia Children Hospital<br>Kemos, Polychronis; Barts and The London School of Medicine and Dentistry Blizard Institute, Centre for Immunobiology<br>Tindemans, Irma; Erasmus University Medical Center-Sophia Children's Hospital, Laboratory of Pediatrics, division of Gastroenterology and Nutrition<br>Aloi, Marina; Sapienza University of Rome, Paediatric Gastroenterology and Liver Unit<br>Koletzko, Sibylle; Dr von Haunersches Kinderspital Kinderklinik und Kinderpoliklinik der Ludwig Maximilian Universitat Munchen, Department of Pediatrics; University of Warmia and Mazury in Olsztyn, Collegium Medicum<br>Levine, Arie; Edith Wolfson Medical Center, Paediatric Gastroenterology and Nutrition Unit; Tel Aviv University<br>Turner, Dan; Shaare Zedek Medical Center, The Hebrew University of Jerusalem, Institute of Paediatric Gastroenterology<br>Veereman, Gigi; UZ Brussel, Department of Paediatric Gastroenterology and Nutrition; Vrije Universiteit Brussel<br>Neyt , Mattias ; ME-TA Medical Evaluation and Technology Assessment<br>russell, richard; Royal Hospital for Children Glasgow, Department of Paediatric Gastroenterology, Hepatology and Nutrition<br>Walters, Thomas D; SickKids Hospital, IBD Centre, Department of Paediatrics<br>Ruemmele, Frank; Hôpital universitaire Necker-Enfants malades, Service de Gastroentérologie pédiatrique; Université Paris Descartes<br>Samsom, Janneke N; Erasmus University Medical Center-Sophia Children's Hospital, Laboratory of Pediatrics<br>Croft, Nicholas; Barts and The London School of Medicine and Dentistry Blizard Institute, Centre for Immunobiology; Queen Mary University of London<br>de Ridder, Lissy; Erasmus MC Sophia Children Hospital, Paediatric gastroenterology
**Primary Subject Heading**:	Gastroenterology and hepatology
Secondary Subject Heading:	Paediatrics

Keywords:	Inflammatory bowel disease < GASTROENTEROLOGY, Paediatric gastroenterology < GASTROENTEROLOGY, IMMUNOLOGY

SCHOLARONE™ Manuscripts

I, the Submitting Author has the right to grant and does grant on behalf of all authors of the Work (as defined in the below author licence), an exclusive licence and/or a non-exclusive licence for contributions from authors who are: i) UK Crown employees; ii) where BMJ has agreed a CC-BY licence shall apply, and/or iii) in accordance with the terms applicable for US Federal Government officers or employees acting as part of their official duties; on a worldwide, perpetual, irrevocable, royalty-free basis to BMJ Publishing Group Ltd ("BMJ") its licensees and where the relevant Journal is co-owned by BMJ to the co-owners of the Journal, to publish the Work in this journal and any other BMJ products and to exploit all rights, as set out in our licence.

The Submitting Author accepts and understands that any supply made under these terms is made by BMJ to the Submitting Author unless you are acting as an employee on behalf of your employer or a postgraduate student of an affiliated institution which is paying any applicable article publishing charge ("APC") for Open Access articles. Where the Submitting Author wishes to make the Work available on an Open Access basis (and intends to pay the relevant APC), the terms of reuse of such Open Access shall be governed by a Creative Commons licence – details of these licences and which Creative Commons licence will apply to this Work are set out in our licence referred to above.

Other than as permitted in any relevant BMJ Author's Self Archiving Policies, I confirm this Work has not been accepted for publication elsewhere, is not being considered for publication elsewhere and does not duplicate material already published. I confirm all authors consent to publication of this Work and authorise the granting of this licence.

For peer review only

Title: An international prospective observational study investigating the disease course and heterogeneity of paediatric-onset inflammatory bowel disease: the protocol of the PIBD-SETQuality inception cohort study

Martine A Aardoom[1], Polychronis Kemos[2], Irma Tindemans[3], Marina Aloi[4], Sibylle Koletzko[5,6,] Arie Levine[7], Dan Turner[8], Gigi Veereman[9], Mattias Neyt[10], Richard K Russell[11], Thomas D Walters[12], Frank M Ruemmele[13], Janneke N Samsom[3], Nicholas M Croft*[2], Lissy de Ridder*[1]; on behalf of the PIBD-SETQuality consortium and PIBD-NET

*These authors are equal senior authors.

Author's affiliations

[1]Department of Paediatric Gastroenterology, The Erasmus MC Sophia Children's Hospital, Rotterdam, the Netherlands;

[2]Paediatric Gastroenterology, Centre for Immunobiology, Blizard Institute, Barts and the London School of Medicine, Queen Mary University of London, London, United Kingdom;

[3]Laboratory of Pediatrics, division Gastroenterology and Nutrition, Erasmus University Medical Center-Sophia Children's Hospital, Rotterdam, the Netherlands;

[4]Paediatric Gastroenterology and Liver Unit, Department of Paediatrics, Sapienza University of Rome, Rome, Italy

[5]Department of Pediatrics, Dr. von Hauner Children's Hospital, University Hospital, LMU Munich, Munich, Germany

[6]Department of Pediatrics, Gastroenterology and Nutrition, School of Medicine Collegium Medicum University of Warmia and Mazury, Olsztyn, Poland

[7]Paediatric Gastroenterology and Nutrition Unit, Tel Aviv University, Edith Wolfson Medical Center, Holon, Israel

For peer review only - http://bmjopen.bmj.com/site/about/guidelines.xhtml
For peer review only

[8]Institute of Paediatric Gastroenterology, Shaare Zedek Medical Center, The Hebrew University of Jerusalem, Jerusalem, Israel

[9]Department of Paediatric Gastroenterology and Nutrition, UZ Brussel – Vrije Universiteit Brussel, Brussels, Belgium

[10]ME-TA Medical Evaluation and Technology Assessment, Merendree, Belgium

[11]Department of Paediatric Gastroenterology, Hepatology and Nutrition, Royal Hospital for Children, Glasgow, United Kingdom

[12]IBD Centre, Department of Paediatrics, SickKids Hospital, University of Toronto, Toronto, Ontario, Canada;

[13]Department of Pediatric Gastroenterology, Université Paris Descartes, Sorbonne Paris Cité, APHP, Hôpital Necker Enfants Malades, Paris, France;

Corresponding author: Lissy de Ridder, MD, PhD

Address: P.O. Box 2060, 3000 CB Rotterdam, the Netherlands

E-mail: l.deridder@erasmusmc.nl, Telephone number: +31 107036049

Word count: 3604

For peer review only

For peer review only - http://bmjopen.bmj.com/site/about/guidelines.xhtml

ABSTRACT

Introduction: Patients with paediatric-onset inflammatory bowel disease (PIBD) may develop a complicated disease course, including growth failure, bowel resection at young age and treatment-related adverse events, all of which can have significant and lasting effects on the patient's development and quality of life. Unfortunately, we are still not able to fully explain the heterogeneity between patients and their disease course and predict which patients will respond to certain therapies or are most at risk of developing a more complicated disease course. To investigate this, large prospective studies with long term follow-up are needed. Currently, no such European or Asian international cohorts exist. In this international cohort, we aim to evaluate disease course and which patients are most at risk of therapy non-response or development of complicated disease based on patient and disease characteristics, immune pathology, and environmental and socio-economic factors.

Methods and analysis: In this international prospective observational study, which is part of the PIBD Network for Safety, Efficacy, Treatment and Quality improvement of care (PIBD-SETQuality), children diagnosed with IBD <18 years are included at diagnosis. The follow-up schedule is in line with standard PIBD care and is intended to continue up to 20 years. Patient and disease characteristics, as well as results of investigations, are collected at baseline and during follow-up. In addition, environmental factors are being assessed (e.g., parent's smoking behaviour, dietary factors and antibiotic use). In specific centres with the ability to perform extensive immunological analyses, blood samples and intestinal biopsies are being collected and analysed (flow cytometry, plasma proteomics, mRNA expression and immunohistochemistry) in therapy naïve patients and during follow-up.

Ethics and dissemination: This study is being conducted according to the principles of the Declaration of Helsinki. Medical-ethical approval has been or will be obtained prior to patient recruitment for all sites.

Trial registration number: NCT03571373

For peer review only - http://bmjopen.bmj.com/site/about/guidelines.xhtml

Keywords: inflammatory bowel disease, Crohn's disease, ulcerative colitis, paediatrics, immunology

Word count abstract (250 – 300): 300

For peer review only

For peer review only - http://bmjopen.bmj.com/site/about/guidelines.xhtml

ARTICLE SUMMARY

Strengths and limitations of this study

Strengths

- International prospective observational study with long-term follow-up examining clinical phenotype and biomarkers that may be predictive for complicated disease course, serious adverse events and response (or not) to therapy.
- The ability to perform extensive immunological analysis (e.g. flow cytometry, plasma proteomics, mRNA expression and immunohistochemistry) of peripheral blood leucocytes and biopsies in therapy-naïve patients to evaluate heterogeneity at diagnosis and to follow-up these immunological parameters during disease course and therapy use.
- The first prospective PIBD cohort that includes patients from several European and some Asian countries, which enables comparison of therapeutic strategies between countries and continents, and outcomes of these strategies.
- Large scale-collection of quality of life measures (e.g. IMPACT III and EQ-5D questionnaires) to correlate to clinical findings and compare this across countries and ethnicities.

Limitations

- Different methods between centres for measurement of laboratory parameters that are part of standard clinical care serve as a limitation to this study.

For peer review only

INTRODUCTION

Background

Paediatric-onset inflammatory bowel disease (PIBD) is a chronic disease that often leads to disabling symptoms such as abdominal pain, diarrhoea and rectal bleeding. Inflammatory bowel disease (IBD) comprises Crohn's disease (CD), ulcerative colitis (UC) and IBD-unclassified (IBD-U). Although IBD is most frequently known as an adult disease, in 5-25% of cases it is diagnosed during childhood or adolescence.[1-3] The incidence of IBD in Western Europe and North-America ranges from 1.85 to 23.82 per 100,000 for CD and 1.9 to 23.14 per 100,000 for UC.[4] Despite the fact that the incidence of IBD varies among different countries, a current concern is that the general trend shows increasing incidence rates over recent decades, especially among the patients <10 years of age.[1 3 5-7] Compared to adult-onset IBD, PIBD reflects a more severe disease.[8-10] Consequences of the disease, such as growth failure and bowel resection at a young age, may have a large impact on the patient's further development and quality of life. In addition, the early onset of this disease regularly leads to an early use of intensive therapies with a life-long risk of treatment-related adverse events.[8 11-13]

The pathogenesis of PIBD is currently partly explained by a combination of a genetic predisposition, microbial factors and susceptibility of the immune system leading to an aberrant inflammatory immune response.[14-16] Treatment strategies therefore focus on modulating or suppressing the immune response using immunosuppressive drugs or biologicals. Despite the known heterogeneity within both paediatric CD and UC patients at disease diagnosis and during follow-up, we are still not able to predict which patients are at risk of developing a complicated disease course and which patients will respond to therapy.[17 18] Therefore, the majority of PIBD patients have been treated following a step-up approach, a strategy in which patients start with a simpler easily available therapy at the bottom of the therapeutic pyramid and medications at the top are often considered more efficacious but may present greater risk to the patient. This approach may lead to a delay in treatment response and increases the risk of ongoing inflammation risking penetrating and stricturing complications. The ability to predict complicated

For peer review only - http://bmjopen.bmj.com/site/about/guidelines.xhtml

disease course and response or non-response to therapy would be of immense value. This is essential to develop strategies that balance, on an individual basis, therapeutic effectiveness with risks of treatment.

In studies including adult IBD patients, several clinical risk factors have been identified for the development of a complicated disease course in Crohn's disease and the need for colectomy in UC patients.[19 20] Given the differences in disease phenotype, course of disease and benefits and risks of treatment between children and adults, findings from adult studies do not directly apply to PIBD. Few studies have assessed risk factors in paediatric-onset CD patients and found that both stricturing disease behaviour[17 21 22] and older age at diagnosis [23-25] are associated with an increased risk for the need for surgery. In paediatric-onset UC patients the Paediatric Ulcerative Colitis Activity Index (PUCAI) at diagnosis and 3 months after diagnosis is found to be an essential predictor of colectomy.[26 27] Kugathasan et al. demonstrated that early anti-TNF therapy in CD was associated with a decreased risk of penetrating but not stricturing complications.[28]

Due to the small number of available studies comprising a large variety of data on several different outcomes and predictors combined with a mainly retrospective set-up, to date most findings regarding predictors of disease course in PIBD are inconclusive. The majority of the identified predictors are demographic or associated with the disease phenotype and studies lack findings on the predictive value of biomarkers. Despite the known role of the immune system in PIBD, no immunological biomarkers have been identified to correlate with the disease phenotype or disease course. Therefore, there is an urgent need to generate a prospective long-term real-world cohort designed to analyse effectiveness and safety signals with the ability to correlate them to individual risk factors in well-phenotyped patients. To address this issue, the Paediatric Inflammatory Bowel Diseases Network for Safety, Efficacy, Treatment and Quality (PIBD-SETQuality) inception cohort was designed. Besides a few prospective PIBD cohorts, established in the United States and Canada, no international European cohorts currently exist to assess this.[28] Due to possible differences in genotype, environmental influences and

For peer review only - http://bmjopen.bmj.com/site/about/guidelines.xhtml

treatment algorithms, European data are required. With this paper, we aim to inform the IBD research community about the existence of the PIBD-SETQuality inception cohort and provide insight into the establishment of this cohort.

Objectives

PIBD-SETQuality is an international project with the overall goal to develop and validate a treatment algorithm for PIBD based on high or low-risk predictors for early complicated or relapsing disease. In this inception cohort, predictors of disease course are identified through prospective collection of longitudinal PIBD data in the first European cohort. Table 1 depicts the potentially relevant prognostic factors listed by the consortium, which formed the basis of parameters to investigate in this study.

Potentially relevant prognostic factors	
**1**	Family history
**2**	Medical history
**3**	Ethnicity
**4**	Severity of disease at diagnosis
**5**	Disease localization
**6**	Course of disease
**7**	Level of inflammatory markers
**8**	Faecal calprotectin level
**9**	Endoscopic findings
**10**	Immunological biomarkers

For peer review only - http://bmjopen.bmj.com/site/about/guidelines.xhtml

**11**	Genetic polymorphisms
**12**	Environmental factors
**13**	Dietary factors
**14**	Health economic status
**15**	Psychosocial status

Table 1. Potentially relevant prognostic factors in prediction of disease course and therapy responsiveness in PIBD

The main objective of this study is to facilitate the discovery of predictors of disease course, treatment response or non-response and severe adverse events in patients with PIBD, by:

1) Collecting real-world longitudinal data with preferably a 20-year follow-up period
2) Collecting biomaterials and linking this to the detailed clinical data
3) Using standardized questionnaires to assess quality of life.

In addition, this cohort enables investigation of PIBD heterogeneity based on immunological biomarkers and racial or environmental factors. Patients of differing ethnicities in the European countries will be included along with patients from some non-European countries, which can allow comparison of different races in immigrant and non-immigrant subgroups.

For peer review only - http://bmjopen.bmj.com/site/about/guidelines.xhtml

METHODS AND ANALYSIS

Study design

The PIBD-SETQuality inception cohort is a multicentre prospective observational study in patients with PIBD. Participants are followed from the moment of diagnosis up to 20 years thereafter. During the first year after diagnosis data collection is performed more frequently and reduced to annual visits after the second year of follow-up (Table 2).

For peer review only

For peer review only - http://bmjopen.bmj.com/site/about/guidelines.xhtml

Visit number	Visit 0	Visit 1	Visit 2	Visit 3	Visit 4	Visit 5	Annual visits	Unscheduled visit
**Time point**	**Prior to start of therapy**	**4 weeks after start of therapy**	**3 months after start of therapy**	**6 months after start of therapy**	**12 months after start of therapy**	**18 months after start of therapy**	**24, 36 months, etc.**	
**Activity**								
Study explained informed consent	X							
Collection of routine clinical and laboratory data, including faecal calprotectin	X	X	X	X	X	X	X	X
Extra blood sample taken at time of routine blood draw (maximum of 20 ml)*	O	O	O	O	O	O	O	O
Extra biopsies taken at time of clinically required colonoscopy (maximum of 8)*	O							O
Tissue sample in case of indication for surgical resection*		O	O	O	O	O	O	O
Environmental questionnaire	X							
IMPACT III & EQ-5D questionnaires	X		X (EQ-5D)	X (EQ-5D)	X (both)	X (EQ-5D)	X (both)	
School attendance and WPAI questionnaires	X (both)		X (both)		X (both)		X (both)	

Table 2. Visit schedule and included activities for PIBD-SETQ inception cohort.
*These activities will only be performed in patients included in the subcohort. ''X'' is performed in all patients, ''O'' is only performed in subcohort patient

For peer review only - http://bmjopen.bmj.com/site/about/guidelines.xhtml

At least 535 PIBD patients need to be included in the inception cohort. However, since this is an observational study, depending on the feasibility, the study will aim to recruit 1000 patients. In specific centres with the capacity to perform immunological analyses participants are included in a subcohort for additional collection of biological specimens. This allows in-depth characterization of immunological pathways in 100 CD patients and 50 UC patients, and provides the opportunity to relate predictive factors to underlying immune dysfunction during follow-up. The inception cohort is part of the PIBD-SETQuality project, which is funded by Horizon 2020.

Eligibility criteria

Children and adolescents <18 years with a likely or confirmed diagnosis of IBD are eligible. Diagnosis has to be made or confirmed within the first 2 months after inclusion. Diagnosis must be based on history, physical examination, laboratory, endoscopic, radiological and histological features according to the revised Porto criteria.[29] If a diagnosis of IBD is not confirmed after the investigations are complete the patient will be excluded from follow-up. Other inclusion criteria comprise available data on all diagnostic procedures for inclusion in the database, informed consent of patient and parents according to the national guidelines, and, in case of inclusion in the subcohort for collection of biomaterial, the patient has not started therapy yet at the moment of diagnostic endoscopy. Patients are excluded from this study 1) when they are on similar treatments as for IBD but for other conditions, defined as the use of any biological, immunosuppressant or systemic corticosteroid, 2) when they are known to have conditions directly affecting IBD (e.g. immunodeficiency or major gastrointestinal resections) and 3) in case of inability to read or understand the patient and family information sheets.

Recruitment and data collection

All eligible patients are asked for informed consent to participate in the inception cohort. Within this study, clinical data and results of questionnaires will be collected in all patients. Biomaterial

For peer review only - http://bmjopen.bmj.com/site/about/guidelines.xhtml

is collected as part of the subcohort in specific centres with the ability to perform immunological analyses.

Clinical data

At baseline, data on demographics, family history, diagnosis, disease activity, disease localization and results of physical examination, endoscopy, radiographic imaging and laboratory results, including faecal calprotectin levels, are collected. Validated scores and classifications such as the weighted Paediatric Crohn's Disease Activity Index (wPCDAI), PUCAI, Simple Endoscopic Score for Crohn's Disease (SES-CD) and Ulcerative Colitis Endoscopic Index of Severity (UCEIS) are used.[30-33] During follow-up, the clinical disease activity scores, results of additional investigations and detailed treatment information are collected at fixed time points and hospitalisations.

Questionnaires

To assess the quality of life (QoL) the validated IMPACT III questionnaire is being used. This questionnaire is a disease specific health-related assessment of QoL, divided in the domains emotional functioning, social functioning, body image, and well-being.[34] [35] Validated EQ-5D questionnaires are being used to assess the health status of the patient (EQ-5D-Y and EQ-5D-Y proxy) and the parents (EQ-5D-5L). The questionnaires consist of five dimensions; mobility, self-care, usual activities, pain/discomfort and anxiety/depression.[36] Parent's work productivity is assessed by the Work Productivity and Activity Impairment questionnaire (WPAI) for caregivers. All validated questionnaires are used in accordance with their respective instructions regarding age limits. If validated translations of the IMPACT III, EQ-5D and WPAI are not available in a certain language, patients in this country will not complete these questionnaires. Due to the lack of validated questionnaires assessing the child's school attendance, a non-validated questionnaire is being used to address this subject. The frequency of the assessment of these questionnaires is shown in Table 2. At baseline, many environmental factors are being assessed comprising e.g. parent's smoking behaviour, antibiotic use, sun exposure, previous enteritis and

For peer review only - http://bmjopen.bmj.com/site/about/guidelines.xhtml

appendectomy. Assessment of dietary factors is limited to assessing 1) whether children are following a vegetarian or vegan diet and 2) if they are excluding specific items from their diet.

Biomaterial

In several centres biological specimens are collected in addition to the clinical data. When informed consent for the collection of biomaterial is obtained, blood samples and intestinal biopsies are collected prior to treatment initiation and during follow-up visits in concurrence with routine clinical diagnostics. At diagnosis, in all patients, biopsies are collected from affected and non-affected tissue in both ileum and colon according to the standard operating procedure written for this study (supplemental Figure 1). During follow-up endoscopies, collection of ileum biopsies is optional and based on the clinician's decision in patients with UC. Analyses of biopsies include immunohistochemistry, targeted quantitative messenger ribonucleic acid (mRNA) analyses and unbiased mRNA sequencing analyses. Peripheral blood samples are collected for plasma proteomics analyses, plasma antimicrobial reactivity, in-depth phenotypic and functional leukocyte analyses and screening for genetic polymorphisms (Figure 1). Immunological analyses will be related to the previously described clinical data that is being collected. Patients enrolled in the subcohort in whom the diagnosis of IBD is not confirmed after additional investigations will be analysed as non-IBD controls.

Outcome measures

This study design and statistical analysis plan are based on one primary and several secondary and exploratory outcomes. At baseline, prior to the start of therapy, the clinical characteristics of PIBD patients will be reviewed and compared by country, as well as racial background. In patients participating in the subcohort, immunological biomarkers are reviewed in order to classify disease heterogeneity based on underlying immune pathology. Thereto, immunological parameters will be related to clinical and morphological disease features at this time point.

For peer review only - http://bmjopen.bmj.com/site/about/guidelines.xhtml

The primary outcome is clinical remission at one year, which reflects our aim to study therapy response and non-response. Clinical remission is defined as a wPCDAI <12.5 in CD patients and a PUCAI <10 in UC or IBDU patients. According to the set hypothesis, the disease localization, extension, behaviour and type of induction therapy are important factors that influence the primary outcome while age at diagnosis and the initial disease activity score are expected to be important covariates. In addition, biochemical and immunological parameters at baseline will be evaluated to predict clinical remission. In a sub-analysis, the effect of early use of immunomodulators and biologics (less than 3 months from diagnosis) on remission rates will be investigated by comparing groups with early and later use.

One of the secondary outcomes of this study is to assess clinical remission over a period of 3 and 6 months since the start of treatment to evaluate short-term response. In addition, the need for treatment intensification within one year will be assessed. The longitudinal follow-up analyses of individual immunological biomarkers will reveal which immune parameters change when patients are in clinical remission. Clinical, biochemical and immunological findings at baseline and 12 weeks after the start of therapy will be assessed for their predictive value on the disease course after one, three, five, 10, 15 and 20 years of follow-up. Lastly, as a longer-term secondary outcome we will assess 1) moderate or severe disease over the last 6-12 months, 2) the development of complications such as fibrostricturing disease, penetrating disease, active perianal fistula or an abscess, 3) the need for IBD-related luminal surgery, and 4) the need for biologic use or need for treatment intensification, after one, three, five, 10, 15 and 20 years.

As an exploratory outcome, the initial management of newly diagnosed PIBD patients will be evaluated. The type of induction therapy and proportion of patients started on early immunomodulators or anti-TNF agents will be assessed, and compared by country or region. Several environmental factors will be evaluated to assess their possible role in the heterogeneity of the presenting phenotype as well as disease course. Lastly, longitudinal findings on QoL, health status, parents' work productivity and child's school attendance will be evaluated, compared by country or region and correlated to disease activity and disease course.

For peer review only

For peer review only - http://bmjopen.bmj.com/site/about/guidelines.xhtml

Sample size calculation

To ensure that the study is adequately powered for the primary outcome, we have calculated the minimum required sample size, which we have also adjusted for a 10% expected information loss and multiple comparisons. To maintain the global significance level alpha at 0.05, for the sample size calculations, we decreased the significance level for the univariate analyses to 0.01 per Bonferroni correction. As we aim to capture predictors with significant influence on the remission, we set the effect size of the primary outcome at 25%. In order to detect a 25% or higher difference between the compared groups, as defined by disease characteristics and therapy induction types and considering the variable sampling ratio (2:1 to 1:2 depending on the factor), we will need 203 CD patients and 214 UC/IBDU patients. Considering approximately 40% of PIBD diagnoses comprises UC/IBDU, we will need 535 PIBD patients. Subsequently, the CD group will have slightly increased power than the originally planned 80%.

Data collection and management

Data are collected in REDCap, a secured database, by using online case report forms (eCRF). CRFs are based on the dataset of the Canadian inception cohort by the Canadian Children Inflammatory Bowel Disease Network (CIDsCaNN) and adjusted to the needs of this study. A monitor performs remote (digital) monitoring for each participating centre yearly after inclusion. The coordinating investigator runs consistency checks on a monthly basis and produces queries to be resolved by the local investigators. To secure accurate comparison of biomarkers in tissue specimens obtained from different centres, highly reproducible sample preparation is required across all centres participating in this study. Therefore, all aspects of sample acquisition and all reagents will be strictly regulated and sample quality will be tightly monitored.

Analysis and statistical methods

For peer review only - http://bmjopen.bmj.com/site/about/guidelines.xhtml

Data analysis will be performed with SPSS version 26 or higher for Windows (IBB SPSS, Chicago, Illinois, USA) and R version 3.6 or higher (R Foundation for Statistical Computing, Vienna, Austria). Descriptive statistics will be computed overall and per disease (CD and UC). Phenotypic grouping will be performed according to specific disease characteristics (e.g. age of onset, disease localization, disease behaviour). Next to this, patients will be categorised according to treatment categories. The appropriate descriptive statistics will be used to summarize the demographic characteristics. The primary outcome will be analysed using a 2-proportion Z test for the comparison of the different groups in our sample, using a stricter confidence level of 0.01 per Bonferroni correction. All proportions will be summarised per group and 95% confidence intervals will be provided. The important factors from the univariate analysis will be used to build a generalised linear model with a logit link function. This will be a multiple analysis of the effects that the selected factors and covariates have on the outcome of clinical remission. The final model will also include interaction terms, if necessary, and will be optimised based on fit diagnostics and residual analysis. The same approach will be used for the secondary outcomes of clinical remission. We will use a mixed-effects linear model to study the effects of the predictors on the disease activity index over time, taking into account the non-independence between the observations due to the repeated measurements from each patient. For the additional long-term secondary outcomes that have a time-to-event nature, we will use Kaplan-Meier curves to summarise the effects of the categorical factors on the outcome. This will be used to build a Cox-regression model with the important factors and covariates that have a significant effect on the time and frequency of the events as defined in the outcomes section. Similar methods will be used for the analysis of the exploratory outcomes. For these outcomes, propensity scores and multivariate methods are required, in particular for the immunological data, including principal component, factor and cluster analysis. Incidences of SAEs and the SAE rate per 100 patient-years will be calculated. Missing data analysis will be performed based on the missing at random (MAR) or missing completely at random (MCAR) mechanisms. Emerging patterns will be thoroughly examined.

For peer review only - http://bmjopen.bmj.com/site/about/guidelines.xhtml

Study status

The first study participant was recruited in 2017. The number of included patients up until February 2020 is 400. Enrolment is expected to be completed by the end of 2021.

Patient and Public Involvement

Patients were not involved in the initial development of this study; however, the French patient charity AFA Crohn, RCH, France, was involved in the final study design and critically reviewed and commented upon main aspects of the trial.

Discussion

The PIBD-SETQuality inception cohort is a unique study, being the first cohort in its size including PIBD patients from European and Asian countries. The homogeneous collection of data from several different countries in one cohort enables comparison of disease phenotype and treatment paradigms between countries and continents. The hypothesised role of environmental factors in the pathogenesis of PIBD, might thus be assessed within this cohort. In addition, being a cohort with real-world data, this study will complement data derived from clinical trials and provide insight in drug use in everyday practice and the related international differences.

The main outcome of this study will be the prediction of a complicated disease course and response or non-response to therapy. One of the important strengths of this study is the collection of biomaterial in therapy naïve patients. Studies assessing immunological biomarkers in PIBD patients are scarce, and even lacking in therapy naïve patients. The prospective set-up of this study will reveal whether immunological biomarkers in PIBD change overtime and in response to therapy. In addition, these biomarkers can be correlated to the clinical data. Besides finding predictive factors of a complicated disease course and response to therapy

For peer review only

within this real-world cohort, we will also use this cohort to examine and possibly validate previously reported findings from other studies such as the GROWTH CD study.[37 38]

ETHICS AND DISSEMINATION

The study is being conducted according to the principles of the Declaration of Helsinki and so far has been approved by the Medical Ethics Committee Erasmus Medical Center of Rotterdam, the National Health Service (NHS) Research Authorities (for England and Wales) and the NHS Research Permission Co-ordinating Centre (Scotland) in the United Kingdom, the Ethical Committee of Ludwig-Maximillians Universität München, the Dubai Healthcare City Authority – Regulatory, the University Malaya Medical Center Medical Ethics Committee, the Ethical Committee of National Center for Child Health and Development in Tokyo, the Committee of Protection of Persons Nord-Ouest I in France, the Institutional Review Board Rabin Medical Center in Israel, the Helsinki Committee of E. Wolfson Medical Center in Israel, Shaare Zedek Medical Center Ethics Committee in Israel, the Ethical Committee Messina, the Regional Ethical Committee Delle Marche in Ancona, the Ethical Committee Universita Degli Studi Della Campania ''Luigi Vanvitelli'' – Azienda Ospedaliera universitaria ''Luigi Vanvitelli'' – A.O.R.N. ''Ospedali Dei Colli'' in Caserta, the Ethical Committee Università Federico II in Naples and the Ethical Committee Unico Regionale in Rome. Handling of patient material complies with the legislation of the relevant country and the European General Data Protection Regulation (GDPR). In accordance with the H2020 general grant agreement, the dissemination process will ensure open access to the peer-reviewed scientific publications resulting from this project and the related bibliographic meta-data.

ACKNOWLEDGEMENTS

We would like to thank all patients who participate in this study; everyone who is participating in data entry and all the gastroenterologists who are involved in patient enrolment. Collaborating

For peer review only - http://bmjopen.bmj.com/site/about/guidelines.xhtml

sites and principal investigators ordered by country:

United Kingdom: N. Croft, Royal London Children's Hospital, Barts Health NHS Trust London; F. Cameron, Alder Hey Children's NHS Foundation, Liverpool; P. Henderson, Royal Hospital for Sick Children, Edinburgh; J. Ashton, University Hospital Southampton NHS Foundation Trust, Southampton; R. Russell, The Royal Hospital for Children Glasgow, Glasgow; M. Rafeeq, Birmingham children's hospital, Birmingham; N. Nedelkopoulou and P. Rao, Sheffield Children's Hospital, Sheffield; A. Rodrigues, Oxford Children's Hospital, Oxford; J. Fell, Chelsea and Westminster Hospital NHS Foundation Trust, London; D. Devadason, Nottingham Children's Hospital, Nottingham; J. Hart, Royal Devon & Exeter NHS Foundation Trust, Exeter; J. Cohen, University College London Hospitals NHS Foundation Trust, London;

The Netherlands: L. de Ridder, Erasmus Medical Center, Rotterdam; T. Hummel, Medisch Spectrum Twente, Enschede; M. Wessels, Rijnstate, Arnhem; C. van der Feen, Jeroen Bosch hospital; 's Hertogenbosch;

Israel: A. Levine, Wolfson Medical Center, Holon; D. Turner, Shaare Zedek Medical Center, Jerusalem; A. Assa, Schneider Children's Medical Center of Israel, Petach-Tikva

Italy: M. Aloi, Sapienza University, Rome;

France: F. Ruemmele, Hôpital Necker Enfants Malades, Paris;

Japan: K. Arai, National Center for Child Health and Development, Tokyo;

Malaysia: WS Lee, University Malay Medical Center, Kuala Lumpur;

United Arabic Emirates: C. Tzivinikos, Al Jalila Children's Specialty Hospital, Dubai

Germany: S. Koletzko, Dr. von Hauner Children's Hospital, Munich.

AUTHOR'S CONTRIBUTIONS

MAA prepared the draft manuscript with comments and review from all authors. MAA, PK, IT, MA, SK, AL, DT, GV, MN, RKR, TW, FMR, JNS, NMC and LdR were involved in the conception, design, planning and drafting of the original research protocol. All authors are involved in the

For peer review only - http://bmjopen.bmj.com/site/about/guidelines.xhtml

implementation of this study. All authors provided critical review of the manuscript and approved the final version.

FUNDING STATEMENT

This project is a Pediatric Inflammatory Bowel Disease Network (PIBD-NET) initiative. It was supported as part of the PIBD-SETQuality project funded by the European Commission Horizon 2020, funding source number 668023. The study sponsor is PIBD-NET.

COMPETING INTERESTS STATEMENT

MA received consultation fee and honorarium from Abbvie. SK received consultation fee, research grant, or honorarium from Danone, Nestec-Nutrition, Abbvie, Takeda, Celgene, Shire, Pfizer, Biogaia, Janssen, Berlin-Chemie, Mead Johnson, Vifor, Pharmacosmos and ThermoFisher. RKR is supported by an NHS Research Scotland Senior Research Fellowship, and has received speakers's fees, travel support, and/or participated in medical board meetings with Nestle, MSD Immunology, AbbVie, Dr Falk, Takeda, Napp, Mead Johnson, Nutricia &4D Pharma. FMR has received speaker fees from Shering-Plough, Nestlé, MeadJohnson, Ferring, MSD, Johnson & Johnson, Centocor, AbbVie; has served as a board member for SAC:DEVELOP (Johnson & Johnson), CAPE (AbbVie), LEA (AbbVie); and has been invited to MSD France, Nestlé Nutrition Institute, Nestlé Health Science, Danone, MeadJohnson, Takeda, Celgene, Biogen, Shire, Pfizer, and Therakos. NMC (into employer's investigator accounts) received speaker fees, advisory board fees, research funding from Eli-Lilly, Takeda, Abbvie Shire Pfizer and 4D Pharma. LdR had collaborations (such as involved in industry-sponsored studies, investigator-initiated study, consultancy) with Shire, Malinckrodt, Nestlé, Celltrion, Abbvie and Pfizer; and received a grant from ZonMw, ECCO and Pfizer. **Remaining authors:** no competing interests declared.

For peer review only - http://bmjopen.bmj.com/site/about/guidelines.xhtml

FIGURE LEGEND

Figure 1. Different immunological analyses that will be performed. Data will be correlated to the clinical data. Abbreviations: IHC, immunohistochemistry; ISH, in situ hybridization.

Supplemental Figure 1. Flow chart depicting the standardized approach for collection of biopsies during the endoscopy.

For peer review only

For peer review only - http://bmjopen.bmj.com/site/about/guidelines.xhtml

REFERENCES

1. Benchimol EI, Fortinsky KJ, Gozdyra P, et al. Epidemiology of pediatric inflammatory bowel disease: a systematic review of international trends. *Inflamm Bowel Dis* 2011;**17**(1):423-39.
2. Gower-Rousseau C, Vasseur F, Fumery M, et al. Epidemiology of inflammatory bowel diseases: new insights from a French population-based registry (EPIMAD). *Dig Liver Dis* 2013;**45**(2):89-94.
3. Ghione S, Sarter H, Fumery M, et al. Dramatic Increase in Incidence of Ulcerative Colitis and Crohn's Disease (1988-2011): A Population-Based Study of French Adolescents. *Am J Gastroenterol* 2018;**113**(2):265-72.
4. Ng SC, Shi HY, Hamidi N, et al. Worldwide incidence and prevalence of inflammatory bowel disease in the 21st century: a systematic review of population-based studies. *Lancet* 2018;**390**(10114):2769-78.
5. Coughlan A, Wylde R, Lafferty L, et al. A rising incidence and poorer male outcomes characterise early onset paediatric inflammatory bowel disease. *Aliment Pharmacol Ther* 2017;**45**(12):1534-41.
6. Bequet E, Sarter H, Fumery M, et al. Incidence and Phenotype at Diagnosis of Very-early-onset Compared with Later-onset Paediatric Inflammatory Bowel Disease: A Population-based Study [1988-2011]. *J Crohns Colitis* 2017;**11**(5):519-26.
7. Sykora J, Pomahacova R, Kreslova M, et al. Current global trends in the incidence of pediatric-onset inflammatory bowel disease. *World J Gastroenterol* 2018;**24**(25):2741-63.
8. Duricova D, Burisch J, Jess T, et al. Age-related differences in presentation and course of inflammatory bowel disease: an update on the population-based literature. *J Crohns Colitis* 2014;**8**(11):1351-61.
9. Duricova D, Fumery M, Annese V, et al. The natural history of Crohn's disease in children: a review of population-based studies. *Eur J Gastroenterol Hepatol* 2017;**29**(2):125-34.
10. Van Limbergen J, Russell RK, Drummond HE, et al. Definition of phenotypic characteristics of childhood-onset inflammatory bowel disease. *Gastroenterology* 2008;**135**(4):1114-22.
11. Singh H, Nugent Z, Targownik LE, et al. Health Care Use by a Population-Based Cohort of Children With Inflammatory Bowel Disease. *Clin Gastroenterol Hepatol* 2015;**13**(7):1302-09 e3.
12. Muller KE, Lakatos PL, Arato A, et al. Incidence, Paris classification, and follow-up in a nationwide incident cohort of pediatric patients with inflammatory bowel disease. *J Pediatr Gastroenterol Nutr* 2013;**57**(5):576-82.
13. Carroll MW, Kuenzig ME, Mack DR, et al. The Impact of Inflammatory Bowel Disease in Canada 2018: Children and Adolescents with IBD. *J Can Assoc Gastroenterol* 2019;**2**(Suppl 1):S49-S67.
14. Kaser A, Zeissig S, Blumberg RS. Inflammatory bowel disease. *Annu Rev Immunol* 2010;**28**:573-621.
15. Jostins L, Ripke S, Weersma RK, et al. Host-microbe interactions have shaped the genetic architecture of inflammatory bowel disease. *Nature* 2012;**491**(7422):119-24.
16. Henderson P, van Limbergen JE, Wilson DC, et al. Genetics of childhood-onset inflammatory bowel disease. *Inflamm Bowel Dis* 2011;**17**(1):346-61.
17. Vernier-Massouille G, Balde M, Salleron J, et al. Natural history of pediatric Crohn's disease: a population-based cohort study. *Gastroenterology* 2008;**135**(4):1106-13.
18. Gower-Rousseau C, Dauchet L, Vernier-Massouille G, et al. The natural history of pediatric ulcerative colitis: a population-based cohort study. *Am J Gastroenterol* 2009;**104**(8):2080-8.
19. Torres J, Caprioli F, Katsanos KH, et al. Predicting Outcomes to Optimize Disease Management in Inflammatory Bowel Diseases. *J Crohns Colitis* 2016;**10**(12):1385-94.
20. Lakatos PL. Prediction of disease course in inflammatory bowel diseases. *World J Gastroenterol* 2010;**16**(21):2589-90.
21. De Greef E, Mahachie John JM, Hoffman I, et al. Profile of pediatric Crohn's disease in Belgium. *J Crohns Colitis* 2013;**7**(11):e588-98.

For peer review only - http://bmjopen.bmj.com/site/about/guidelines.xhtml

22. Rinawi F, Assa A, Hartman C, et al. Incidence of Bowel Surgery and Associated Risk Factors in Pediatric-Onset Crohn's Disease. *Inflamm Bowel Dis* 2016;**22**(12):2917-23.
23. Gupta N, Cohen SA, Bostrom AG, et al. Risk factors for initial surgery in pediatric patients with Crohn's disease. *Gastroenterology* 2006;**130**(4):1069-77.
24. Henderson P, Kennedy NA, Van Limbergen JE, et al. Serum C-reactive protein and CRP genotype in pediatric inflammatory bowel disease: influence on phenotype, natural history, and response to therapy. *Inflamm Bowel Dis* 2015;**21**(3):596-605.
25. Schaefer ME, Machan JT, Kawatu D, et al. Factors that determine risk for surgery in pediatric patients with Crohn's disease. *Clin Gastroenterol Hepatol* 2010;**8**(9):789-94.
26. Rinawi F, Assa A, Eliakim R, et al. Risk of Colectomy in Patients With Pediatric-onset Ulcerative Colitis. *J Pediatr Gastroenterol Nutr* 2017;**65**(4):410-15.
27. Schechter A, Griffiths C, Gana JC, et al. Early endoscopic, laboratory and clinical predictors of poor disease course in paediatric ulcerative colitis. *Gut* 2015;**64**(4):580-8.
28. Kugathasan S, Denson LA, Walters TD, et al. Prediction of complicated disease course for children newly diagnosed with Crohn's disease: a multicentre inception cohort study. *Lancet* 2017;**389**(10080):1710-18.
29. Levine A, Koletzko S, Turner D, et al. ESPGHAN revised porto criteria for the diagnosis of inflammatory bowel disease in children and adolescents. *J Pediatr Gastroenterol Nutr* 2014;**58**(6):795-806.
30. Turner D, Otley AR, Mack D, et al. Development, validation, and evaluation of a pediatric ulcerative colitis activity index: a prospective multicenter study. *Gastroenterology* 2007;**133**(2):423-32.
31. Hyams J, Markowitz J, Otley A, et al. Evaluation of the pediatric crohn disease activity index: a prospective multicenter experience. *J Pediatr Gastroenterol Nutr* 2005;**41**(4):416-21.
32. Daperno M, D'Haens G, Van Assche G, et al. Development and validation of a new, simplified endoscopic activity score for Crohn's disease: the SES-CD. *Gastrointest Endosc* 2004;**60**(4):505-12.
33. Travis SP, Schnell D, Krzeski P, et al. Developing an instrument to assess the endoscopic severity of ulcerative colitis: the Ulcerative Colitis Endoscopic Index of Severity (UCEIS). *Gut* 2012;**61**(4):535-42.
34. Werner H, Landolt MA, Buehr P, et al. Validation of the IMPACT-III quality of life questionnaire in Swiss children with inflammatory bowel disease. *J Crohns Colitis* 2014;**8**(7):641-8.
35. Abdovic S, Mocic Pavic A, Milosevic M, et al. The IMPACT-III (HR) questionnaire: a valid measure of health-related quality of life in Croatian children with inflammatory bowel disease. *J Crohns Colitis* 2013;**7**(11):908-15.
36. Herdman M, Gudex C, Lloyd A, et al. Development and preliminary testing of the new five-level version of EQ-5D (EQ-5D-5L). *Qual Life Res* 2011;**20**(10):1727-36.
37. Ziv-Baran T, Hussey S, Sladek M, et al. Response to treatment is more important than disease severity at diagnosis for prediction of early relapse in new-onset paediatric Crohn's disease. *Aliment Pharmacol Ther* 2018;**48**(11-12):1242-50.
38. Levine A, Chanchlani N, Hussey S, et al. Complicated Disease and Response to Initial Therapy Predicts Early Surgery in Paediatric Crohn's Disease: Results From the Porto Group GROWTH Study. *J Crohns Colitis* 2020;**14**(1):71-78.

For peer review only - http://bmjopen.bmj.com/site/about/guidelines.xhtml

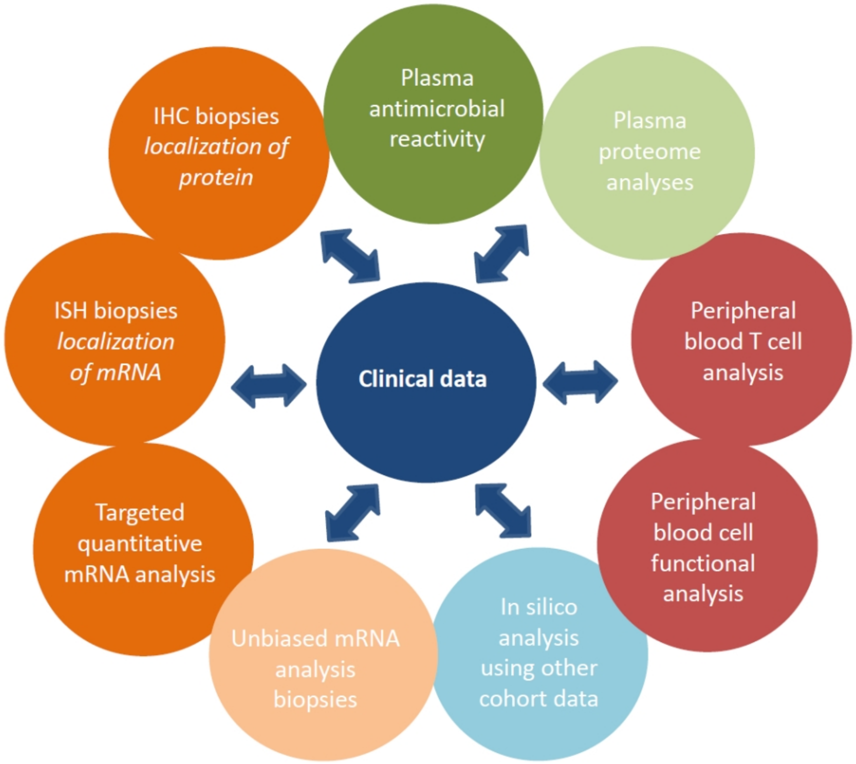

Different immunological analyses that will be performed. Data will be correlated to the clinical data. Abbreviations: IHC, immunohistochemistry; ISH, in situ hybridization.

PIBD-SETQuality Inception cohort: Flow chart biopsies

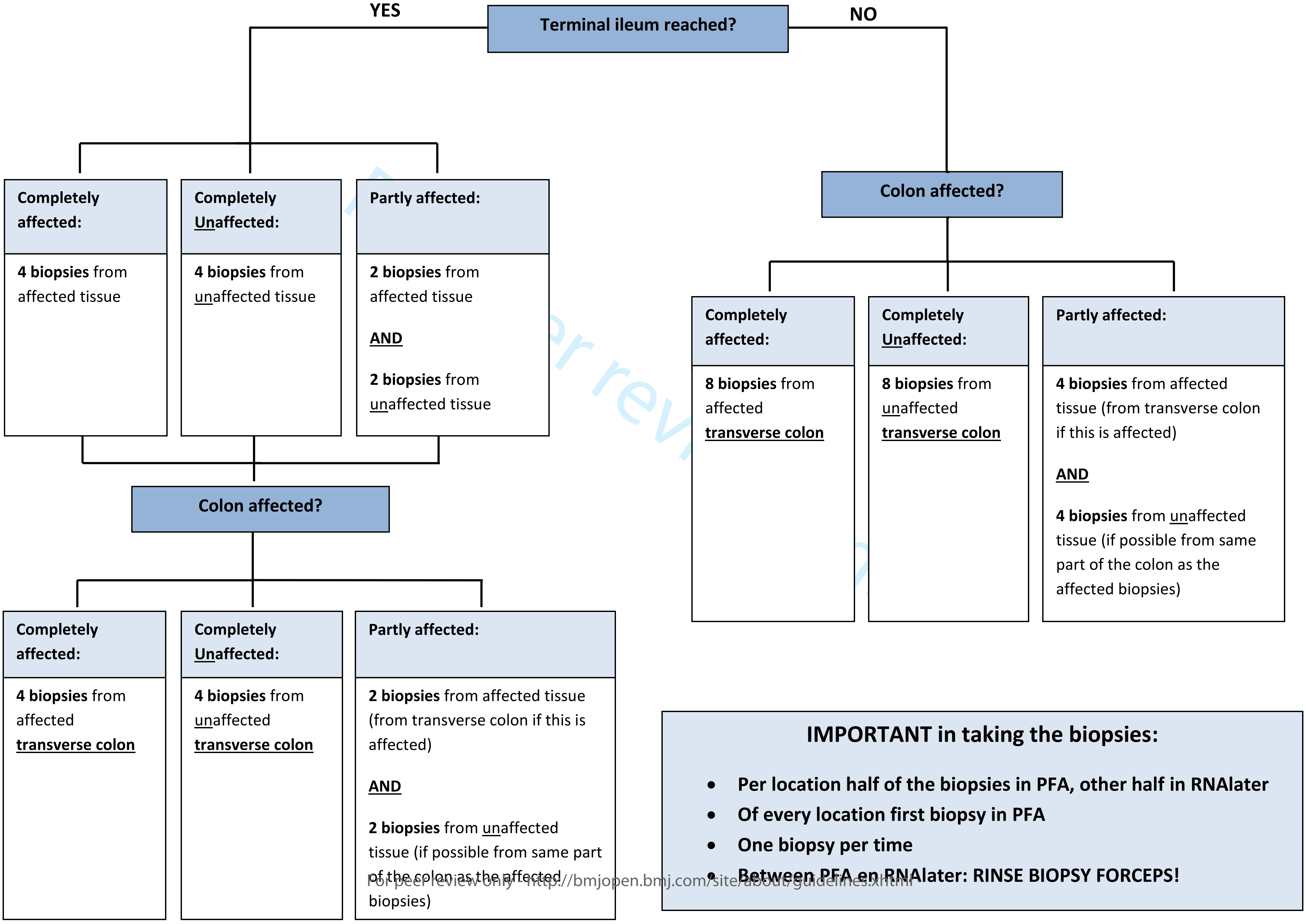

For peer review only - http://bmjopen.bmj.com/site/about/guidelines.xhtml

STROBE 2007 (v4) Statement—Checklist of items that should be included in reports of *cohort studies*

Section/Topic	Item #	Recommendation	Reported on page #
**Title and abstract**	1	(*a*) Indicate the study's design with a commonly used term in the title or the abstract	1
		(*b*) Provide in the abstract an informative and balanced summary of what was done and what was found	3
**Introduction**			
Background/rationale	2	Explain the scientific background and rationale for the investigation being reported	6-8
Objectives	3	State specific objectives, including any prespecified hypotheses	8-9
**Methods**			
Study design	4	Present key elements of study design early in the paper	10
Setting	5	Describe the setting, locations, and relevant dates, including periods of recruitment, exposure, follow-up, and data collection	9-13
Participants	6	(*a*) Give the eligibility criteria, and the sources and methods of selection of participants. Describe methods of follow-up	11
		(*b*) For matched studies, give matching criteria and number of exposed and unexposed	NA
Variables	7	Clearly define all outcomes, exposures, predictors, potential confounders, and effect modifiers. Give diagnostic criteria, if applicable	13-15
Data sources/ measurement	8*	For each variable of interest, give sources of data and details of methods of assessment (measurement). Describe comparability of assessment methods if there is more than one group	13-17
Bias	9	Describe any efforts to address potential sources of bias	16-17
Study size	10	Explain how the study size was arrived at	15
Quantitative variables	11	Explain how quantitative variables were handled in the analyses. If applicable, describe which groupings were chosen and why	16
Statistical methods	12	(*a*) Describe all statistical methods, including those used to control for confounding	16-17
		(*b*) Describe any methods used to examine subgroups and interactions	16
		(*c*) Explain how missing data were addressed	16-17
		(*d*) If applicable, explain how loss to follow-up was addressed	16-17
		(*e*) Describe any sensitivity analyses	16
**Results**			

For peer review only

Participants	13*	(a) Report numbers of individuals at each stage of study—eg numbers potentially eligible, examined for eligibility, confirmed eligible, included in the study, completing follow-up, and analysed	n/a
		(b) Give reasons for non-participation at each stage	n/a
		(c) Consider use of a flow diagram	n/a
Descriptive data	14*	(a) Give characteristics of study participants (eg demographic, clinical, social) and information on exposures and potential confounders	n/a
		(b) Indicate number of participants with missing data for each variable of interest	n/a
		(c) Summarise follow-up time (eg, average and total amount)	n/a
Outcome data	15*	Report numbers of outcome events or summary measures over time	n/a
Main results	16	(*a*) Give unadjusted estimates and, if applicable, confounder-adjusted estimates and their precision (eg, 95% confidence interval). Make clear which confounders were adjusted for and why they were included	n/a
		(*b*) Report category boundaries when continuous variables were categorized	n/a
		(*c*) If relevant, consider translating estimates of relative risk into absolute risk for a meaningful time period	n/a
Other analyses	17	Report other analyses done—eg analyses of subgroups and interactions, and sensitivity analyses	n/a
**Discussion**			
Key results	18	Summarise key results with reference to study objectives	n/a
**Limitations**			
Interpretation	20	Give a cautious overall interpretation of results considering objectives, limitations, multiplicity of analyses, results from similar studies, and other relevant evidence	n/a
Generalisability	21	Discuss the generalisability (external validity) of the study results	n/a
**Other information**			
Funding	22	Give the source of funding and the role of the funders for the present study and, if applicable, for the original study on which the present article is based	20

*Give information separately for cases and controls in case-control studies and, if applicable, for exposed and unexposed groups in cohort and cross-sectional studies.

Note: An Explanation and Elaboration article discusses each checklist item and gives methodological background and published examples of transparent reporting. The STROBE checklist is best used in conjunction with this article (freely available on the Web sites of PLoS Medicine at http://www.plosmedicine.org/, Annals of Internal Medicine at http://www.annals.org/, and Epidemiology at http://www.epidem.com/). Information on the STROBE Initiative is available at www.strobe-statement.org.

For peer review only

BMJ Open

An international prospective observational study investigating the disease course and heterogeneity of paediatric-onset inflammatory bowel disease: the protocol of the PIBD-SETQuality inception cohort study

Journal:	*BMJ Open*
Manuscript ID	bmjopen-2019-035538.R2
Article Type:	Protocol
Date Submitted by the Author:	23-Apr-2020
Complete List of Authors:	Aardoom, Martine A; Erasmus MC Sophia Children Hospital<br>Kemos, Polychronis; Barts and The London School of Medicine and Dentistry Blizard Institute, Centre for Immunobiology<br>Tindemans, Irma; Erasmus University Medical Center-Sophia Children's Hospital, Laboratory of Pediatrics, division of Gastroenterology and Nutrition<br>Aloi, Marina; Sapienza University of Rome, Paediatric Gastroenterology and Liver Unit<br>Koletzko, Sibylle; Dr von Haunersches Kinderspital Kinderklinik und Kinderpoliklinik der Ludwig Maximilian Universitat Munchen, Department of Pediatrics; University of Warmia and Mazury in Olsztyn, Collegium Medicum<br>Levine, Arie; Edith Wolfson Medical Center, Paediatric Gastroenterology and Nutrition Unit; Tel Aviv University<br>Turner, Dan; Shaare Zedek Medical Center, The Hebrew University of Jerusalem, Institute of Paediatric Gastroenterology<br>Veereman, Gigi; UZ Brussel, Department of Paediatric Gastroenterology and Nutrition; Vrije Universiteit Brussel<br>Neyt , Mattias ; ME-TA Medical Evaluation and Technology Assessment<br>russell, richard; Royal Hospital for Children Glasgow, Department of Paediatric Gastroenterology, Hepatology and Nutrition<br>Walters, Thomas D; SickKids Hospital, IBD Centre, Department of Paediatrics<br>Ruemmele, Frank; Hôpital universitaire Necker-Enfants malades, Service de Gastroentérologie pédiatrique; Université Paris Descartes<br>Samsom, Janneke N; Erasmus University Medical Center-Sophia Children's Hospital, Laboratory of Pediatrics<br>Croft, Nicholas; Barts and The London School of Medicine and Dentistry Blizard Institute, Centre for Immunobiology; Queen Mary University of London<br>de Ridder, Lissy; Erasmus MC Sophia Children Hospital, Paediatric gastroenterology
**Primary Subject Heading**:	Gastroenterology and hepatology
Secondary Subject Heading:	Paediatrics

Keywords:	Inflammatory bowel disease < GASTROENTEROLOGY, Paediatric gastroenterology < GASTROENTEROLOGY, IMMUNOLOGY

SCHOLARONE™
Manuscripts

I, the Submitting Author has the right to grant and does grant on behalf of all authors of the Work (as defined in the below author licence), an exclusive licence and/or a non-exclusive licence for contributions from authors who are: i) UK Crown employees; ii) where BMJ has agreed a CC-BY licence shall apply, and/or iii) in accordance with the terms applicable for US Federal Government officers or employees acting as part of their official duties; on a worldwide, perpetual, irrevocable, royalty-free basis to BMJ Publishing Group Ltd ("BMJ") its licensees and where the relevant Journal is co-owned by BMJ to the co-owners of the Journal, to publish the Work in this journal and any other BMJ products and to exploit all rights, as set out in our licence.

The Submitting Author accepts and understands that any supply made under these terms is made by BMJ to the Submitting Author unless you are acting as an employee on behalf of your employer or a postgraduate student of an affiliated institution which is paying any applicable article publishing charge ("APC") for Open Access articles. Where the Submitting Author wishes to make the Work available on an Open Access basis (and intends to pay the relevant APC), the terms of reuse of such Open Access shall be governed by a Creative Commons licence – details of these licences and which Creative Commons licence will apply to this Work are set out in our licence referred to above.

Other than as permitted in any relevant BMJ Author's Self Archiving Policies, I confirm this Work has not been accepted for publication elsewhere, is not being considered for publication elsewhere and does not duplicate material already published. I confirm all authors consent to publication of this Work and authorise the granting of this licence.

For peer review only

Title: An international prospective observational study investigating the disease course and heterogeneity of paediatric-onset inflammatory bowel disease: the protocol of the PIBD-SETQuality inception cohort study

Martine A Aardoom[1], Polychronis Kemos[2], Irma Tindemans[3], Marina Aloi[4], Sibylle Koletzko[5,6,] Arie Levine[7], Dan Turner[8], Gigi Veereman[9], Mattias Neyt[10], Richard K Russell[11], Thomas D Walters[12], Frank M Ruemmele[13], Janneke N Samsom[3], Nicholas M Croft*[2], Lissy de Ridder*[1]; on behalf of the PIBD-SETQuality consortium and PIBD-NET

*These authors are equal senior authors.

Author's affiliations

[1]Department of Paediatric Gastroenterology, The Erasmus MC Sophia Children's Hospital, Rotterdam, the Netherlands;

[2]Paediatric Gastroenterology, Centre for Immunobiology, Blizard Institute, Barts and the London School of Medicine, Queen Mary University of London, London, United Kingdom;

[3]Laboratory of Pediatrics, division Gastroenterology and Nutrition, Erasmus University Medical Center-Sophia Children's Hospital, Rotterdam, the Netherlands;

[4]Paediatric Gastroenterology and Liver Unit, Department of Paediatrics, Sapienza University of Rome, Rome, Italy

[5]Department of Pediatrics, Dr. von Hauner Children's Hospital, University Hospital, LMU Munich, Munich, Germany

[6]Department of Pediatrics, Gastroenterology and Nutrition, School of Medicine Collegium Medicum University of Warmia and Mazury, Olsztyn, Poland

[7]Paediatric Gastroenterology and Nutrition Unit, Tel Aviv University, Edith Wolfson Medical Center, Holon, Israel

For peer review only

For peer review only - http://bmjopen.bmj.com/site/about/guidelines.xhtml

[8]Institute of Paediatric Gastroenterology, Shaare Zedek Medical Center, The Hebrew University of Jerusalem, Jerusalem, Israel

[9]Department of Paediatric Gastroenterology and Nutrition, UZ Brussel – Vrije Universiteit Brussel, Brussels, Belgium

[10]ME-TA Medical Evaluation and Technology Assessment, Merendree, Belgium

[11]Department of Paediatric Gastroenterology, Hepatology and Nutrition, Royal Hospital for Children, Glasgow, United Kingdom

[12]IBD Centre, Department of Paediatrics, SickKids Hospital, University of Toronto, Toronto, Ontario, Canada;

[13]Department of Pediatric Gastroenterology, Université Paris Descartes, Sorbonne Paris Cité, APHP, Hôpital Necker Enfants Malades, Paris, France;

Corresponding author: Lissy de Ridder, MD, PhD

Address: P.O. Box 2060, 3000 CB Rotterdam, the Netherlands

E-mail: l.deridder@erasmusmc.nl, Telephone number: +31 107036049

Word count: 3604

For peer review only

For peer review only - http://bmjopen.bmj.com/site/about/guidelines.xhtml

ABSTRACT

Introduction: Patients with paediatric-onset inflammatory bowel disease (PIBD) may develop a complicated disease course, including growth failure, bowel resection at young age and treatment-related adverse events, all of which can have significant and lasting effects on the patient's development and quality of life. Unfortunately, we are still not able to fully explain the heterogeneity between patients and their disease course and predict which patients will respond to certain therapies or are most at risk of developing a more complicated disease course. To investigate this, large prospective studies with long term follow-up are needed. Currently, no such European or Asian international cohorts exist. In this international cohort, we aim to evaluate disease course and which patients are most at risk of therapy non-response or development of complicated disease based on patient and disease characteristics, immune pathology, and environmental and socio-economic factors.

Methods and analysis: In this international prospective observational study, which is part of the PIBD Network for Safety, Efficacy, Treatment and Quality improvement of care (PIBD-SETQuality), children diagnosed with IBD <18 years are included at diagnosis. The follow-up schedule is in line with standard PIBD care and is intended to continue up to 20 years. Patient and disease characteristics, as well as results of investigations, are collected at baseline and during follow-up. In addition, environmental factors are being assessed (e.g., parent's smoking behaviour, dietary factors and antibiotic use). In specific centres with the ability to perform extensive immunological analyses, blood samples and intestinal biopsies are being collected and analysed (flow cytometry, plasma proteomics, mRNA expression and immunohistochemistry) in therapy naïve patients and during follow-up.

Ethics and dissemination: Medical-ethical approval has been obtained prior to patient recruitment for all sites. The results will be disseminated through peer-reviewed scientific publications.

Trial registration number: NCT03571373

For peer review only - http://bmjopen.bmj.com/site/about/guidelines.xhtml

Keywords: inflammatory bowel disease, Crohn's disease, ulcerative colitis, paediatrics, immunology

Word count abstract (250 – 300): 300

For peer review only

For peer review only - http://bmjopen.bmj.com/site/about/guidelines.xhtml

ARTICLE SUMMARY

Strengths and limitations of this study

Strengths

- International prospective observational study with long-term follow-up examining clinical phenotype and biomarkers that may be predictive for complicated disease course, serious adverse events and response (or not) to therapy.
- The ability to perform extensive immunological analysis (e.g. flow cytometry, plasma proteomics, mRNA expression and immunohistochemistry) of peripheral blood leucocytes and biopsies in therapy-naïve patients to evaluate heterogeneity at diagnosis and to follow-up these immunological parameters during disease course and therapy use.
- The first prospective PIBD cohort that includes patients from several European and some Asian countries, which enables comparison of therapeutic strategies between countries and continents, and outcomes of these strategies.
- Large scale-collection of quality of life measures (e.g. IMPACT III and EQ-5D questionnaires) to correlate to clinical findings and compare this across countries and ethnicities.

Limitations

- Different methods between centres for measurement of laboratory parameters that are part of standard clinical care serve as a limitation to this study.

For peer review only

For peer review only - http://bmjopen.bmj.com/site/about/guidelines.xhtml

INTRODUCTION

Background

Paediatric-onset inflammatory bowel disease (PIBD) is a chronic disease that often leads to disabling symptoms such as abdominal pain, diarrhoea and rectal bleeding. Inflammatory bowel disease (IBD) comprises Crohn's disease (CD), ulcerative colitis (UC) and IBD-unclassified (IBD-U). Although IBD is most frequently known as an adult disease, in 5-25% of cases it is diagnosed during childhood or adolescence.[1-3] The incidence of IBD in Western Europe and North-America ranges from 1.85 to 23.82 per 100,000 for CD and 1.9 to 23.14 per 100,000 for UC.[4] Despite the fact that the incidence of IBD varies among different countries, a current concern is that the general trend shows increasing incidence rates over recent decades, especially among the patients <10 years of age.[1 3 5-7] Compared to adult-onset IBD, PIBD reflects a more severe disease.[8-10] Consequences of the disease, such as growth failure and bowel resection at a young age, may have a large impact on the patient's further development and quality of life. In addition, the early onset of this disease regularly leads to an early use of intensive therapies with a life-long risk of treatment-related adverse events.[8 11-13]

The pathogenesis of PIBD is currently partly explained by a combination of a genetic predisposition, microbial factors and susceptibility of the immune system leading to an aberrant inflammatory immune response.[14-16] Treatment strategies therefore focus on modulating or suppressing the immune response using immunosuppressive drugs or biologicals. Despite the known heterogeneity within both paediatric CD and UC patients at disease diagnosis and during follow-up, we are still not able to predict which patients are at risk of developing a complicated disease course and which patients will respond to therapy.[17 18] Therefore, the majority of PIBD patients have been treated following a step-up approach, a strategy in which patients start with a simpler easily available therapy at the bottom of the therapeutic pyramid and medications at the top are often considered more efficacious but may present greater risk to the patient. This approach may lead to a delay in treatment response and increases the risk of ongoing inflammation risking penetrating and stricturing complications. The ability to predict complicated

For peer review only - http://bmjopen.bmj.com/site/about/guidelines.xhtml

disease course and response or non-response to therapy would be of immense value. This is essential to develop strategies that balance, on an individual basis, therapeutic effectiveness with risks of treatment.

In studies including adult IBD patients, several clinical risk factors have been identified for the development of a complicated disease course in Crohn's disease and the need for colectomy in UC patients.[19 20] Given the differences in disease phenotype, course of disease and benefits and risks of treatment between children and adults, findings from adult studies do not directly apply to PIBD. Few studies have assessed risk factors in paediatric-onset CD patients and found that both stricturing disease behaviour[17 21 22] and older age at diagnosis [23-25] are associated with an increased risk for the need for surgery. In paediatric-onset UC patients the Paediatric Ulcerative Colitis Activity Index (PUCAI) at diagnosis and 3 months after diagnosis is found to be an essential predictor of colectomy.[26 27] Kugathasan et al. demonstrated that early anti-TNF therapy in CD was associated with a decreased risk of penetrating but not stricturing complications.[28]

Due to the small number of available studies comprising a large variety of data on several different outcomes and predictors combined with a mainly retrospective set-up, to date most findings regarding predictors of disease course in PIBD are inconclusive. The majority of the identified predictors are demographic or associated with the disease phenotype and studies lack findings on the predictive value of biomarkers. Despite the known role of the immune system in PIBD, no immunological biomarkers have been identified to correlate with the disease phenotype or disease course. Therefore, there is an urgent need to generate a prospective long-term real-world cohort designed to analyse effectiveness and safety signals with the ability to correlate them to individual risk factors in well-phenotyped patients. To address this issue, the Paediatric Inflammatory Bowel Diseases Network for Safety, Efficacy, Treatment and Quality (PIBD-SETQuality) inception cohort was designed. Besides a few prospective PIBD cohorts, established in the United States and Canada, no international European cohorts currently exist to assess this.[28] Due to possible differences in genotype, environmental influences and

For peer review only - http://bmjopen.bmj.com/site/about/guidelines.xhtml

treatment algorithms, European data are required. With this paper, we aim to inform the IBD research community about the existence of the PIBD-SETQuality inception cohort and provide insight into the establishment of this cohort.

Objectives

PIBD-SETQuality is an international project with the overall goal to develop and validate a treatment algorithm for PIBD based on high or low-risk predictors for early complicated or relapsing disease. In this inception cohort, predictors of disease course are identified through prospective collection of longitudinal PIBD data in the first cohort that includes patients from several European and some Asian centres. Table 1 depicts the potentially relevant prognostic factors listed by the consortium, which formed the basis of parameters to investigate in this study.

**Potentially relevant prognostic factors**	
**1**	Family history
**2**	Medical history
**3**	Ethnicity
**4**	Severity of disease at diagnosis
**5**	Disease localization
**6**	Course of disease
**7**	Level of inflammatory markers
**8**	Faecal calprotectin level
**9**	Endoscopic findings

For peer review only - http://bmjopen.bmj.com/site/about/guidelines.xhtml

**10**	Immunological biomarkers
**11**	Genetic polymorphisms
**12**	Environmental factors
**13**	Dietary factors
**14**	Health economic status
**15**	Psychosocial status

Table 1. Potentially relevant prognostic factors in prediction of disease course and therapy responsiveness in PIBD

The main objective of this study is to facilitate the discovery of predictors of disease course, treatment response or non-response and severe adverse events in patients with PIBD, by:

1) Collecting real-world longitudinal data with preferably a 20-year follow-up period
2) Collecting biomaterials and linking this to the detailed clinical data
3) Using standardized questionnaires to assess quality of life.

In addition, this cohort enables investigation of PIBD heterogeneity based on immunological biomarkers and racial or environmental factors. Patients of differing ethnicities in the European countries will be included along with patients from some non-European countries, which can allow comparison of different races in immigrant and non-immigrant subgroups.

For peer review only - http://bmjopen.bmj.com/site/about/guidelines.xhtml

METHODS AND ANALYSIS

Study design

The PIBD-SETQuality inception cohort is a multicentre prospective observational study in patients with PIBD. Participants are followed from the moment of diagnosis up to 20 years thereafter. During the first year after diagnosis data collection is performed more frequently and reduced to annual visits after the second year of follow-up (Table 2).

For peer review only

For peer review only - http://bmjopen.bmj.com/site/about/guidelines.xhtml

Visit number	Visit 0	Visit 1	Visit 2	Visit 3	Visit 4	Visit 5	Annual visits	Unscheduled visit
**Time point**	**Prior to start of therapy**	**4 weeks after start of therapy**	**3 months after start of therapy**	**6 months after start of therapy**	**12 months after start of therapy**	**18 months after start of therapy**	**24, 36 months, etc.**	
**Activity**								
Study explained informed consent	X							
Collection of routine clinical and laboratory data, including faecal calprotectin	X	X	X	X	X	X	X	X
Extra blood sample taken at time of routine blood draw (maximum of 20 ml)*	O	O	O	O	O	O	O	O
Extra biopsies taken at time of clinically required colonoscopy (maximum of 8)*	O							O
Tissue sample in case of indication for surgical resection*		O	O	O	O	O	O	O
Environmental questionnaire	X							
IMPACT III & EQ-5D questionnaires	X		X (EQ-5D)	X (EQ-5D)	X (both)	X (EQ-5D)	X (both)	
School attendance and WPAI questionnaires	X (both)		X (both)		X (both)		X (both)	

Table 2. Visit schedule and included activities for PIBD-SETQ inception cohort.
*These activities will only be performed in patients included in the subcohort. ''X'' is performed in all patients, ''O'' is only performed in subcohort patient

For peer review only

For peer review only - http://bmjopen.bmj.com/site/about/guidelines.xhtml

At least 535 PIBD patients need to be included in the inception cohort. However, since this is an observational study, depending on the feasibility, the study will aim to recruit 1000 patients. In specific centres with the capacity to perform immunological analyses participants are included in a subcohort for additional collection of biological specimens. This allows in-depth characterization of immunological pathways in 100 CD patients and 50 UC patients, and provides the opportunity to relate predictive factors to underlying immune dysfunction during follow-up. The inception cohort is part of the PIBD-SETQuality project, which is funded by Horizon 2020.

Eligibility criteria

Children and adolescents <18 years with a likely or confirmed diagnosis of IBD are eligible. Diagnosis has to be made or confirmed within the first 2 months after inclusion. Diagnosis must be based on history, physical examination, laboratory, endoscopic, radiological and histological features according to the revised Porto criteria.[29] If a diagnosis of IBD is not confirmed after the investigations are complete the patient will be excluded from follow-up. Other inclusion criteria comprise available data on all diagnostic procedures for inclusion in the database, informed consent of patient and parents according to the national guidelines, and, in case of inclusion in the subcohort for collection of biomaterial, the patient has not started therapy yet at the moment of diagnostic endoscopy. Patients are excluded from this study 1) when they are on similar treatments as for IBD but for other conditions, defined as the use of any biological, immunosuppressant or systemic corticosteroid, 2) when they are known to have conditions directly affecting IBD (e.g. immunodeficiency or major gastrointestinal resections) and 3) in case of inability to read or understand the patient and family information sheets.

Recruitment and data collection

All eligible patients are asked for informed consent to participate in the inception cohort. Within this study, clinical data and results of questionnaires will be collected in all patients. Biomaterial

For peer review only - http://bmjopen.bmj.com/site/about/guidelines.xhtml

is collected as part of the subcohort in specific centres with the ability to perform immunological analyses.

Clinical data

At baseline, data on demographics, family history, diagnosis, disease activity, disease localization and results of physical examination, endoscopy, radiographic imaging and laboratory results, including faecal calprotectin levels, are collected. Validated scores and classifications such as the weighted Paediatric Crohn's Disease Activity Index (wPCDAI), PUCAI, Simple Endoscopic Score for Crohn's Disease (SES-CD) and Ulcerative Colitis Endoscopic Index of Severity (UCEIS) are used.[30-33] During follow-up, the clinical disease activity scores, results of additional investigations and detailed treatment information are collected at fixed time points and hospitalisations.

Questionnaires

To assess the quality of life (QoL) the validated IMPACT III questionnaire is being used. This questionnaire is a disease specific health-related assessment of QoL, divided in the domains emotional functioning, social functioning, body image, and well-being.[34] [35] Validated EQ-5D questionnaires are being used to assess the health status of the patient (EQ-5D-Y and EQ-5D-Y proxy) and the parents (EQ-5D-5L). The questionnaires consist of five dimensions; mobility, self-care, usual activities, pain/discomfort and anxiety/depression.[36] Parent's work productivity is assessed by the Work Productivity and Activity Impairment questionnaire (WPAI) for caregivers. All validated questionnaires are used in accordance with their respective instructions regarding age limits. If validated translations of the IMPACT III, EQ-5D and WPAI are not available in a certain language, patients in this country will not complete these questionnaires. Due to the lack of validated questionnaires assessing the child's school attendance, a non-validated questionnaire is being used to address this subject. The frequency of the assessment of these questionnaires is shown in Table 2. At baseline, many environmental factors are being assessed comprising e.g. parent's smoking behaviour, antibiotic use, sun exposure, previous enteritis and

For peer review only - http://bmjopen.bmj.com/site/about/guidelines.xhtml

appendectomy. Assessment of dietary factors is limited to assessing 1) whether children are following a vegetarian or vegan diet and 2) if they are excluding specific items from their diet.

Biomaterial

In several centres biological specimens are collected in addition to the clinical data. When informed consent for the collection of biomaterial is obtained, blood samples and intestinal biopsies are collected prior to treatment initiation and during follow-up visits in concurrence with routine clinical diagnostics. At diagnosis, in all patients, biopsies are collected from affected and non-affected tissue in both ileum and colon according to the standard operating procedure written for this study (supplemental Figure 1). During follow-up endoscopies, collection of ileum biopsies is optional and based on the clinician's decision in patients with UC. Analyses of biopsies include immunohistochemistry, targeted quantitative messenger ribonucleic acid (mRNA) analyses and unbiased mRNA sequencing analyses. Peripheral blood samples are collected for plasma proteomics analyses, plasma antimicrobial reactivity, in-depth phenotypic and functional leukocyte analyses and screening for genetic polymorphisms (Figure 1). Immunological analyses will be related to the previously described clinical data that is being collected. Patients enrolled in the subcohort in whom the diagnosis of IBD is not confirmed after additional investigations will be analysed as non-IBD controls.

Outcome measures

This study design and statistical analysis plan are based on one primary and several secondary and exploratory outcomes. At baseline, prior to the start of therapy, the clinical characteristics of PIBD patients will be reviewed and compared by country, as well as racial background. In patients participating in the subcohort, immunological biomarkers are reviewed in order to classify disease heterogeneity based on underlying immune pathology. Thereto, immunological parameters will be related to clinical and morphological disease features at this time point.

For peer review only - http://bmjopen.bmj.com/site/about/guidelines.xhtml

The primary outcome is clinical remission at one year, which reflects our aim to study therapy response and non-response. Clinical remission is defined as a wPCDAI <12.5 in CD patients and a PUCAI <10 in UC or IBDU patients. According to the set hypothesis, the disease localization, extension, behaviour and type of induction therapy are important factors that influence the primary outcome while age at diagnosis and the initial disease activity score are expected to be important covariates. In addition, biochemical and immunological parameters at baseline will be evaluated to predict clinical remission. In a sub-analysis, the effect of early use of immunomodulators and biologics (less than 3 months from diagnosis) on remission rates will be investigated by comparing groups with early and later use.

One of the secondary outcomes of this study is to assess clinical remission over a period of 3 and 6 months since the start of treatment to evaluate short-term response. In addition, the need for treatment intensification within one year will be assessed. The longitudinal follow-up analyses of individual immunological biomarkers will reveal which immune parameters change when patients are in clinical remission. Clinical, biochemical and immunological findings at baseline and 12 weeks after the start of therapy will be assessed for their predictive value on the disease course after one, three, five, 10, 15 and 20 years of follow-up. Lastly, as a longer-term secondary outcome we will assess 1) moderate or severe disease over the last 6-12 months, 2) the development of complications such as fibrostricturing disease, penetrating disease, active perianal fistula or an abscess, 3) the need for IBD-related luminal surgery, and 4) the need for biologic use or need for treatment intensification, after one, three, five, 10, 15 and 20 years.

As an exploratory outcome, the initial management of newly diagnosed PIBD patients will be evaluated. The type of induction therapy and proportion of patients started on early immunomodulators or anti-TNF agents will be assessed, and compared by country or region. Several environmental factors will be evaluated to assess their possible role in the heterogeneity of the presenting phenotype as well as disease course. Lastly, longitudinal findings on QoL, health status, parents' work productivity and child's school attendance will be evaluated, compared by country or region and correlated to disease activity and disease course.

For peer review only - http://bmjopen.bmj.com/site/about/guidelines.xhtml

Sample size calculation

To ensure that the study is adequately powered for the primary outcome, we have calculated the minimum required sample size, which we have also adjusted for a 10% expected information loss and multiple comparisons. To maintain the global significance level alpha at 0.05, for the sample size calculations, we decreased the significance level for the univariate analyses to 0.01 per Bonferroni correction. As we aim to capture predictors with significant influence on the remission, we set the effect size of the primary outcome at 25%. In order to detect a 25% or higher difference between the compared groups, as defined by disease characteristics and therapy induction types and considering the variable sampling ratio (2:1 to 1:2 depending on the factor), we will need 203 CD patients and 214 UC/IBDU patients. Considering approximately 40% of PIBD diagnoses comprises UC/IBDU, we will need 535 PIBD patients. Subsequently, the CD group will have slightly increased power than the originally planned 80%.

Data collection and management

Data are collected in REDCap, a secured database, by using online case report forms (eCRF). CRFs are based on the dataset of the Canadian inception cohort by the Canadian Children Inflammatory Bowel Disease Network (CIDsCaNN) and adjusted to the needs of this study. A monitor performs remote (digital) monitoring for each participating centre yearly after inclusion. The coordinating investigator runs consistency checks on a monthly basis and produces queries to be resolved by the local investigators. To secure accurate comparison of biomarkers in tissue specimens obtained from different centres, highly reproducible sample preparation is required across all centres participating in this study. Therefore, all aspects of sample acquisition and all reagents will be strictly regulated and sample quality will be tightly monitored.

Analysis and statistical methods

For peer review only - http://bmjopen.bmj.com/site/about/guidelines.xhtml

Data analysis will be performed with SPSS version 26 or higher for Windows (IBB SPSS, Chicago, Illinois, USA) and R version 3.6 or higher (R Foundation for Statistical Computing, Vienna, Austria). Descriptive statistics will be computed overall and per disease (CD and UC). Phenotypic grouping will be performed according to specific disease characteristics (e.g. age of onset, disease localization, disease behaviour). Next to this, patients will be categorised according to treatment categories. The appropriate descriptive statistics will be used to summarize the demographic characteristics. The primary outcome will be analysed using a 2-proportion Z test for the comparison of the different groups in our sample, using a stricter confidence level of 0.01 per Bonferroni correction. All proportions will be summarised per group and 95% confidence intervals will be provided. The important factors from the univariate analysis will be used to build a generalised linear model with a logit link function. This will be a multiple analysis of the effects that the selected factors and covariates have on the outcome of clinical remission. The final model will also include interaction terms, if necessary, and will be optimised based on fit diagnostics and residual analysis. The same approach will be used for the secondary outcomes of clinical remission. We will use a mixed-effects linear model to study the effects of the predictors on the disease activity index over time, taking into account the non-independence between the observations due to the repeated measurements from each patient. For the additional long-term secondary outcomes that have a time-to-event nature, we will use Kaplan-Meier curves to summarise the effects of the categorical factors on the outcome. This will be used to build a Cox-regression model with the important factors and covariates that have a significant effect on the time and frequency of the events as defined in the outcomes section. Similar methods will be used for the analysis of the exploratory outcomes. For these outcomes, propensity scores and multivariate methods are required, in particular for the immunological data, including principal component, factor and cluster analysis. Incidences of SAEs and the SAE rate per 100 patient-years will be calculated. Missing data analysis will be performed based on the missing at random (MAR) or missing completely at random (MCAR) mechanisms. Emerging patterns will be thoroughly examined.

For peer review only

For peer review only - http://bmjopen.bmj.com/site/about/guidelines.xhtml

Study status

The first study participant was recruited in 2017. The number of included patients up until February 2020 is 400. Enrolment is expected to be completed by the end of 2021.

Patient and Public Involvement

Patients were not involved in the initial development of this study; however, the French patient charity AFA Crohn, RCH, France, was involved in the final study design and critically reviewed and commented upon main aspects of the trial.

Discussion

The PIBD-SETQuality inception cohort is a unique study, being the first cohort in its size including PIBD patients from European and Asian countries. The homogeneous collection of data from several different countries in one cohort enables comparison of disease phenotype and treatment paradigms between countries and continents. The hypothesised role of environmental factors in the pathogenesis of PIBD, might thus be assessed within this cohort. In addition, being a cohort with real-world data, this study will complement data derived from clinical trials and provide insight in drug use in everyday practice and the related international differences.

The main outcome of this study will be the prediction of a complicated disease course and response or non-response to therapy. One of the important strengths of this study is the collection of biomaterial in therapy naïve patients. Studies assessing immunological biomarkers in PIBD patients are scarce, and even lacking in therapy naïve patients. The prospective set-up of this study will reveal whether immunological biomarkers in PIBD change overtime and in response to therapy. In addition, these biomarkers can be correlated to the clinical data. Besides finding predictive factors of a complicated disease course and response to therapy

For peer review only - http://bmjopen.bmj.com/site/about/guidelines.xhtml

within this real-world cohort, we will also use this cohort to examine and possibly validate previously reported findings from other studies such as the GROWTH CD study.[37 38]

ETHICS AND DISSEMINATION

The study is being conducted according to the principles of the Declaration of Helsinki and so far has been approved by the Medical Ethics Committee Erasmus Medical Center of Rotterdam, the National Health Service (NHS) Research Authorities (for England and Wales) and the NHS Research Permission Co-ordinating Centre (Scotland) in the United Kingdom, the Ethical Committee of Ludwig-Maximillians Universität München, the Dubai Healthcare City Authority – Regulatory, the University Malaya Medical Center Medical Ethics Committee, the Ethical Committee of National Center for Child Health and Development in Tokyo, the Committee of Protection of Persons Nord-Ouest I in France, the Institutional Review Board Rabin Medical Center in Israel, the Helsinki Committee of E. Wolfson Medical Center in Israel, Shaare Zedek Medical Center Ethics Committee in Israel, the Ethical Committee Messina, the Regional Ethical Committee Delle Marche in Ancona, the Ethical Committee Universita Degli Studi Della Campania ''Luigi Vanvitelli'' – Azienda Ospedaliera universitaria ''Luigi Vanvitelli'' – A.O.R.N. ''Ospedali Dei Colli'' in Caserta, the Ethical Committee Università Federico II in Naples and the Ethical Committee Unico Regionale in Rome. Handling of patient material complies with the legislation of the relevant country and the European General Data Protection Regulation (GDPR). In accordance with the H2020 general grant agreement, the dissemination process will ensure open access to the peer-reviewed scientific publications resulting from this project and the related bibliographic meta-data.

ACKNOWLEDGEMENTS

We would like to thank all patients who participate in this study; everyone who is participating in data entry and all the gastroenterologists who are involved in patient enrolment. Collaborating

For peer review only - http://bmjopen.bmj.com/site/about/guidelines.xhtml

sites and principal investigators ordered by country:

United Kingdom: N. Croft, Royal London Children's Hospital, Barts Health NHS Trust London; F. Cameron, Alder Hey Children's NHS Foundation, Liverpool; P. Henderson, Royal Hospital for Sick Children, Edinburgh; J. Ashton, University Hospital Southampton NHS Foundation Trust, Southampton; R. Russell, The Royal Hospital for Children Glasgow, Glasgow; M. Rafeeq, Birmingham children's hospital, Birmingham; N. Nedelkopoulou and P. Rao, Sheffield Children's Hospital, Sheffield; A. Rodrigues, Oxford Children's Hospital, Oxford; J. Fell, Chelsea and Westminster Hospital NHS Foundation Trust, London; D. Devadason, Nottingham Children's Hospital, Nottingham; J. Hart, Royal Devon & Exeter NHS Foundation Trust, Exeter; J. Cohen, University College London Hospitals NHS Foundation Trust, London;

The Netherlands: L. de Ridder, Erasmus Medical Center, Rotterdam; T. Hummel, Medisch Spectrum Twente, Enschede; M. Wessels, Rijnstate, Arnhem; C. van der Feen, Jeroen Bosch hospital; 's Hertogenbosch;

Israel: A. Levine, Wolfson Medical Center, Holon; D. Turner, Shaare Zedek Medical Center, Jerusalem; A. Assa, Schneider Children's Medical Center of Israel, Petach-Tikva

Italy: M. Aloi, Sapienza University, Rome;

France: F. Ruemmele, Hôpital Necker Enfants Malades, Paris;

Japan: K. Arai, National Center for Child Health and Development, Tokyo;

Malaysia: WS Lee, University Malay Medical Center, Kuala Lumpur;

United Arabic Emirates: C. Tzivinikos, Al Jalila Children's Specialty Hospital, Dubai

Germany: S. Koletzko, Dr. von Hauner Children's Hospital, Munich.

AUTHOR'S CONTRIBUTIONS

MAA prepared the draft manuscript with comments and review from all authors. MAA, PK, IT, MA, SK, AL, DT, GV, MN, RKR, TW, FMR, JNS, NMC and LdR were involved in the conception, design, planning and drafting of the original research protocol. All authors are involved in the

For peer review only - http://bmjopen.bmj.com/site/about/guidelines.xhtml

implementation of this study. All authors provided critical review of the manuscript and approved the final version.

FUNDING STATEMENT

This project is a Pediatric Inflammatory Bowel Disease Network (PIBD-NET) initiative. It was supported as part of the PIBD-SETQuality project funded by the European Commission Horizon 2020, funding source number 668023. The study sponsor is PIBD-NET.

COMPETING INTERESTS STATEMENT

MA received consultation fee and honorarium from Abbvie. SK received consultation fee, research grant, or honorarium from Danone, Nestec-Nutrition, Abbvie, Takeda, Celgene, Shire, Pfizer, Biogaia, Janssen, Berlin-Chemie, Mead Johnson, Vifor, Pharmacosmos and ThermoFisher. RKR is supported by an NHS Research Scotland Senior Research Fellowship, and has received speakers's fees, travel support, and/or participated in medical board meetings with Nestle, MSD Immunology, AbbVie, Dr Falk, Takeda, Napp, Mead Johnson, Nutricia &4D Pharma. FMR has received speaker fees from Shering-Plough, Nestlé, MeadJohnson, Ferring, MSD, Johnson & Johnson, Centocor, AbbVie; has served as a board member for SAC:DEVELOP (Johnson & Johnson), CAPE (AbbVie), LEA (AbbVie); and has been invited to MSD France, Nestlé Nutrition Institute, Nestlé Health Science, Danone, MeadJohnson, Takeda, Celgene, Biogen, Shire, Pfizer, and Therakos. NMC (into employer's investigator accounts) received speaker fees, advisory board fees, research funding from Eli-Lilly, Takeda, Abbvie Shire Pfizer and 4D Pharma. LdR had collaborations (such as involved in industry-sponsored studies, investigator-initiated study, consultancy) with Shire, Malinckrodt, Nestlé, Celltrion, Abbvie and Pfizer; and received a grant from ZonMw, ECCO and Pfizer. **Remaining authors:** no competing interests declared.

For peer review only - http://bmjopen.bmj.com/site/about/guidelines.xhtml

FIGURE LEGEND

Figure 1. Different immunological analyses that will be performed. Data will be correlated to the clinical data. Abbreviations: IHC, immunohistochemistry; ISH, in situ hybridization.

Supplemental Figure 1. Flow chart depicting the standardized approach for collection of biopsies during the endoscopy.

For peer review only

REFERENCES

1. Benchimol EI, Fortinsky KJ, Gozdyra P, et al. Epidemiology of pediatric inflammatory bowel disease: a systematic review of international trends. *Inflamm Bowel Dis* 2011;**17**(1):423-39.
2. Gower-Rousseau C, Vasseur F, Fumery M, et al. Epidemiology of inflammatory bowel diseases: new insights from a French population-based registry (EPIMAD). *Dig Liver Dis* 2013;**45**(2):89-94.
3. Ghione S, Sarter H, Fumery M, et al. Dramatic Increase in Incidence of Ulcerative Colitis and Crohn's Disease (1988-2011): A Population-Based Study of French Adolescents. *Am J Gastroenterol* 2018;**113**(2):265-72.
4. Ng SC, Shi HY, Hamidi N, et al. Worldwide incidence and prevalence of inflammatory bowel disease in the 21st century: a systematic review of population-based studies. *Lancet* 2018;**390**(10114):2769-78.
5. Coughlan A, Wylde R, Lafferty L, et al. A rising incidence and poorer male outcomes characterise early onset paediatric inflammatory bowel disease. *Aliment Pharmacol Ther* 2017;**45**(12):1534-41.
6. Bequet E, Sarter H, Fumery M, et al. Incidence and Phenotype at Diagnosis of Very-early-onset Compared with Later-onset Paediatric Inflammatory Bowel Disease: A Population-based Study [1988-2011]. *J Crohns Colitis* 2017;**11**(5):519-26.
7. Sykora J, Pomahacova R, Kreslova M, et al. Current global trends in the incidence of pediatric-onset inflammatory bowel disease. *World J Gastroenterol* 2018;**24**(25):2741-63.
8. Duricova D, Burisch J, Jess T, et al. Age-related differences in presentation and course of inflammatory bowel disease: an update on the population-based literature. *J Crohns Colitis* 2014;**8**(11):1351-61.
9. Duricova D, Fumery M, Annese V, et al. The natural history of Crohn's disease in children: a review of population-based studies. *Eur J Gastroenterol Hepatol* 2017;**29**(2):125-34.
10. Van Limbergen J, Russell RK, Drummond HE, et al. Definition of phenotypic characteristics of childhood-onset inflammatory bowel disease. *Gastroenterology* 2008;**135**(4):1114-22.
11. Singh H, Nugent Z, Targownik LE, et al. Health Care Use by a Population-Based Cohort of Children With Inflammatory Bowel Disease. *Clin Gastroenterol Hepatol* 2015;**13**(7):1302-09 e3.
12. Muller KE, Lakatos PL, Arato A, et al. Incidence, Paris classification, and follow-up in a nationwide incident cohort of pediatric patients with inflammatory bowel disease. *J Pediatr Gastroenterol Nutr* 2013;**57**(5):576-82.
13. Carroll MW, Kuenzig ME, Mack DR, et al. The Impact of Inflammatory Bowel Disease in Canada 2018: Children and Adolescents with IBD. *J Can Assoc Gastroenterol* 2019;**2**(Suppl 1):S49-S67.
14. Kaser A, Zeissig S, Blumberg RS. Inflammatory bowel disease. *Annu Rev Immunol* 2010;**28**:573-621.
15. Jostins L, Ripke S, Weersma RK, et al. Host-microbe interactions have shaped the genetic architecture of inflammatory bowel disease. *Nature* 2012;**491**(7422):119-24.
16. Henderson P, van Limbergen JE, Wilson DC, et al. Genetics of childhood-onset inflammatory bowel disease. *Inflamm Bowel Dis* 2011;**17**(1):346-61.
17. Vernier-Massouille G, Balde M, Salleron J, et al. Natural history of pediatric Crohn's disease: a population-based cohort study. *Gastroenterology* 2008;**135**(4):1106-13.
18. Gower-Rousseau C, Dauchet L, Vernier-Massouille G, et al. The natural history of pediatric ulcerative colitis: a population-based cohort study. *Am J Gastroenterol* 2009;**104**(8):2080-8.
19. Torres J, Caprioli F, Katsanos KH, et al. Predicting Outcomes to Optimize Disease Management in Inflammatory Bowel Diseases. *J Crohns Colitis* 2016;**10**(12):1385-94.
20. Lakatos PL. Prediction of disease course in inflammatory bowel diseases. *World J Gastroenterol* 2010;**16**(21):2589-90.
21. De Greef E, Mahachie John JM, Hoffman I, et al. Profile of pediatric Crohn's disease in Belgium. *J Crohns Colitis* 2013;**7**(11):e588-98.

For peer review only - http://bmjopen.bmj.com/site/about/guidelines.xhtml

22. Rinawi F, Assa A, Hartman C, et al. Incidence of Bowel Surgery and Associated Risk Factors in Pediatric-Onset Crohn's Disease. *Inflamm Bowel Dis* 2016;**22**(12):2917-23.

23. Gupta N, Cohen SA, Bostrom AG, et al. Risk factors for initial surgery in pediatric patients with Crohn's disease. *Gastroenterology* 2006;**130**(4):1069-77.

24. Henderson P, Kennedy NA, Van Limbergen JE, et al. Serum C-reactive protein and CRP genotype in pediatric inflammatory bowel disease: influence on phenotype, natural history, and response to therapy. *Inflamm Bowel Dis* 2015;**21**(3):596-605.

25. Schaefer ME, Machan JT, Kawatu D, et al. Factors that determine risk for surgery in pediatric patients with Crohn's disease. *Clin Gastroenterol Hepatol* 2010;**8**(9):789-94.

26. Rinawi F, Assa A, Eliakim R, et al. Risk of Colectomy in Patients With Pediatric-onset Ulcerative Colitis. *J Pediatr Gastroenterol Nutr* 2017;**65**(4):410-15.

27. Schechter A, Griffiths C, Gana JC, et al. Early endoscopic, laboratory and clinical predictors of poor disease course in paediatric ulcerative colitis. *Gut* 2015;**64**(4):580-8.

28. Kugathasan S, Denson LA, Walters TD, et al. Prediction of complicated disease course for children newly diagnosed with Crohn's disease: a multicentre inception cohort study. *Lancet* 2017;**389**(10080):1710-18.

29. Levine A, Koletzko S, Turner D, et al. ESPGHAN revised porto criteria for the diagnosis of inflammatory bowel disease in children and adolescents. *J Pediatr Gastroenterol Nutr* 2014;**58**(6):795-806.

30. Turner D, Otley AR, Mack D, et al. Development, validation, and evaluation of a pediatric ulcerative colitis activity index: a prospective multicenter study. *Gastroenterology* 2007;**133**(2):423-32.

31. Hyams J, Markowitz J, Otley A, et al. Evaluation of the pediatric crohn disease activity index: a prospective multicenter experience. *J Pediatr Gastroenterol Nutr* 2005;**41**(4):416-21.

32. Daperno M, D'Haens G, Van Assche G, et al. Development and validation of a new, simplified endoscopic activity score for Crohn's disease: the SES-CD. *Gastrointest Endosc* 2004;**60**(4):505-12.

33. Travis SP, Schnell D, Krzeski P, et al. Developing an instrument to assess the endoscopic severity of ulcerative colitis: the Ulcerative Colitis Endoscopic Index of Severity (UCEIS). *Gut* 2012;**61**(4):535-42.

34. Werner H, Landolt MA, Buehr P, et al. Validation of the IMPACT-III quality of life questionnaire in Swiss children with inflammatory bowel disease. *J Crohns Colitis* 2014;**8**(7):641-8.

35. Abdovic S, Mocic Pavic A, Milosevic M, et al. The IMPACT-III (HR) questionnaire: a valid measure of health-related quality of life in Croatian children with inflammatory bowel disease. *J Crohns Colitis* 2013;**7**(11):908-15.

36. Herdman M, Gudex C, Lloyd A, et al. Development and preliminary testing of the new five-level version of EQ-5D (EQ-5D-5L). *Qual Life Res* 2011;**20**(10):1727-36.

37. Ziv-Baran T, Hussey S, Sladek M, et al. Response to treatment is more important than disease severity at diagnosis for prediction of early relapse in new-onset paediatric Crohn's disease. *Aliment Pharmacol Ther* 2018;**48**(11-12):1242-50.

38. Levine A, Chanchlani N, Hussey S, et al. Complicated Disease and Response to Initial Therapy Predicts Early Surgery in Paediatric Crohn's Disease: Results From the Porto Group GROWTH Study. *J Crohns Colitis* 2020;**14**(1):71-78.

For peer review only - http://bmjopen.bmj.com/site/about/guidelines.xhtml

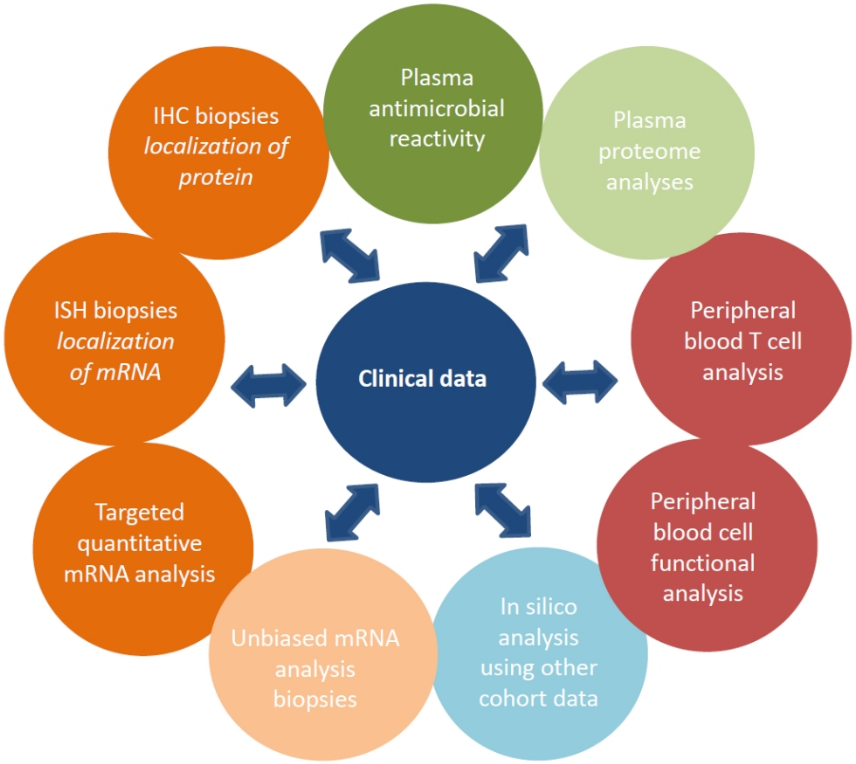

Different immunological analyses that will be performed. Data will be correlated to the clinical data.
Abbreviations: IHC, immunohistochemistry; ISH, in situ hybridization.

PIBD-SETQuality Inception cohort: Flow chart biopsies

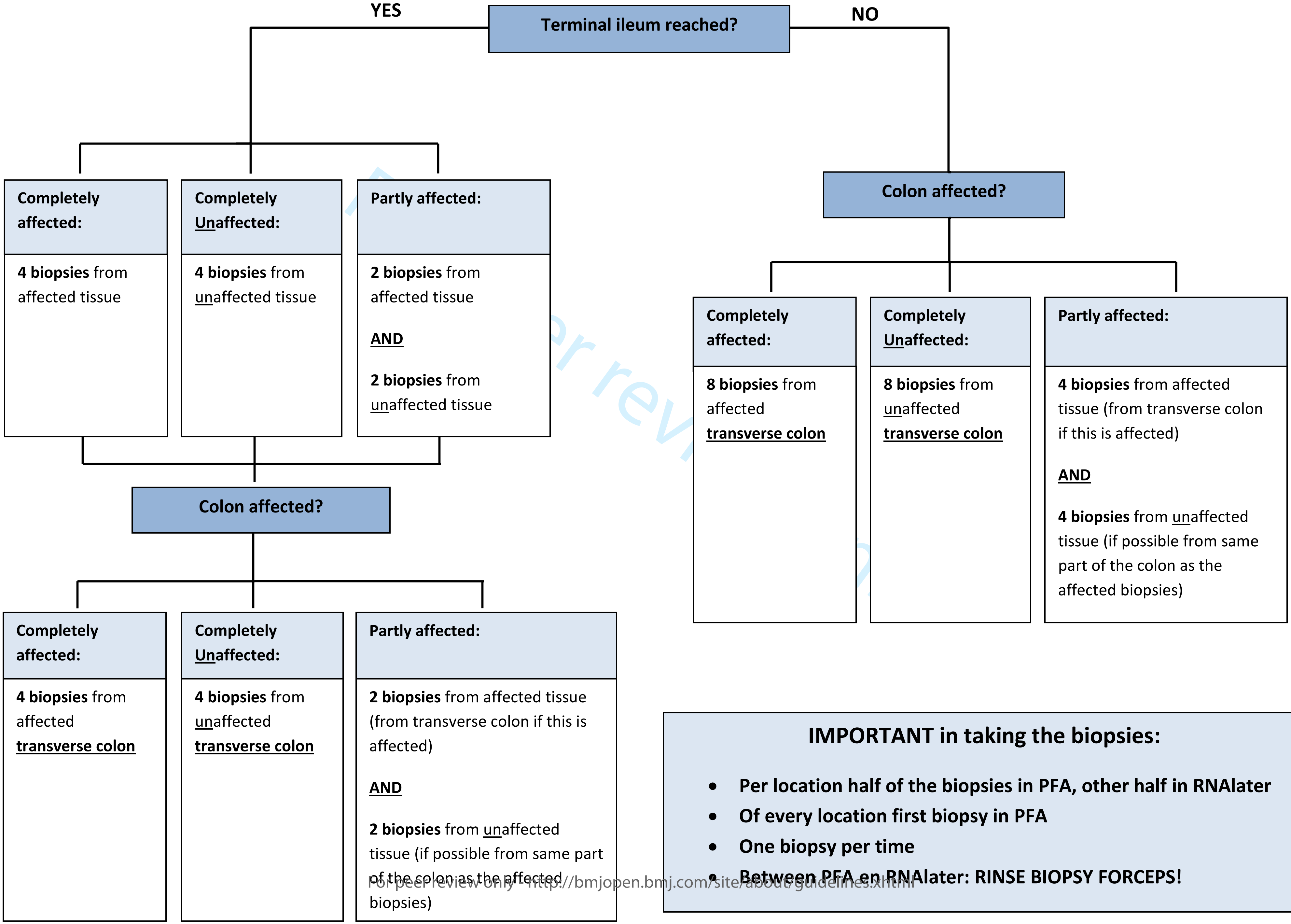

IMPORTANT in taking the biopsies:

- **Per location half of the biopsies in PFA, other half in RNAlater**
- **Of every location first biopsy in PFA**
- **One biopsy per time**
- **Between PFA en RNAlater: RINSE BIOPSY FORCEPS!**

For peer review only - http://bmjopen.bmj.com/site/about/guidelines.xhtml

STROBE 2007 (v4) Statement—Checklist of items that should be included in reports of *cohort studies*

Section/Topic	Item #	Recommendation	Reported on page #
**Title and abstract**	1	(*a*) Indicate the study's design with a commonly used term in the title or the abstract	1
		(*b*) Provide in the abstract an informative and balanced summary of what was done and what was found	3
**Introduction**			
Background/rationale	2	Explain the scientific background and rationale for the investigation being reported	6-8
Objectives	3	State specific objectives, including any prespecified hypotheses	8-9
**Methods**			
Study design	4	Present key elements of study design early in the paper	10
Setting	5	Describe the setting, locations, and relevant dates, including periods of recruitment, exposure, follow-up, and data collection	9-13
Participants	6	(*a*) Give the eligibility criteria, and the sources and methods of selection of participants. Describe methods of follow-up	11
		(*b*) For matched studies, give matching criteria and number of exposed and unexposed	NA
Variables	7	Clearly define all outcomes, exposures, predictors, potential confounders, and effect modifiers. Give diagnostic criteria, if applicable	13-15
Data sources/ measurement	8*	For each variable of interest, give sources of data and details of methods of assessment (measurement). Describe comparability of assessment methods if there is more than one group	13-17
Bias	9	Describe any efforts to address potential sources of bias	16-17
Study size	10	Explain how the study size was arrived at	15
Quantitative variables	11	Explain how quantitative variables were handled in the analyses. If applicable, describe which groupings were chosen and why	16
Statistical methods	12	(*a*) Describe all statistical methods, including those used to control for confounding	16-17
		(*b*) Describe any methods used to examine subgroups and interactions	16
		(*c*) Explain how missing data were addressed	16-17
		(*d*) If applicable, explain how loss to follow-up was addressed	16-17
		(*e*) Describe any sensitivity analyses	16
**Results**			

For peer review only

Participants	13*	(a) Report numbers of individuals at each stage of study—eg numbers potentially eligible, examined for eligibility, confirmed eligible, included in the study, completing follow-up, and analysed	n/a
		(b) Give reasons for non-participation at each stage	n/a
		(c) Consider use of a flow diagram	n/a
Descriptive data	14*	(a) Give characteristics of study participants (eg demographic, clinical, social) and information on exposures and potential confounders	n/a
		(b) Indicate number of participants with missing data for each variable of interest	n/a
		(c) Summarise follow-up time (eg, average and total amount)	n/a
Outcome data	15*	Report numbers of outcome events or summary measures over time	n/a
Main results	16	(*a*) Give unadjusted estimates and, if applicable, confounder-adjusted estimates and their precision (eg, 95% confidence interval). Make clear which confounders were adjusted for and why they were included	n/a
		(*b*) Report category boundaries when continuous variables were categorized	n/a
		(*c*) If relevant, consider translating estimates of relative risk into absolute risk for a meaningful time period	n/a
Other analyses	17	Report other analyses done—eg analyses of subgroups and interactions, and sensitivity analyses	n/a
**Discussion**			
Key results	18	Summarise key results with reference to study objectives	n/a
**Limitations**			
Interpretation	20	Give a cautious overall interpretation of results considering objectives, limitations, multiplicity of analyses, results from similar studies, and other relevant evidence	n/a
Generalisability	21	Discuss the generalisability (external validity) of the study results	n/a
**Other information**			
Funding	22	Give the source of funding and the role of the funders for the present study and, if applicable, for the original study on which the present article is based	20

*Give information separately for cases and controls in case-control studies and, if applicable, for exposed and unexposed groups in cohort and cross-sectional studies.

Note: An Explanation and Elaboration article discusses each checklist item and gives methodological background and published examples of transparent reporting. The STROBE checklist is best used in conjunction with this article (freely available on the Web sites of PLoS Medicine at http://www.plosmedicine.org/, Annals of Internal Medicine at http://www.annals.org/, and Epidemiology at http://www.epidem.com/). Information on the STROBE Initiative is available at www.strobe-statement.org.

For peer review only
